# 5C analysis of the Epidermal Differentiation Complex locus reveals distinct chromatin interaction networks between gene-rich and gene-poor TADs in skin epithelial cells

**DOI:** 10.1371/journal.pgen.1006966

**Published:** 2017-09-01

**Authors:** Krzysztof Poterlowicz, Joanne L. Yarker, Igor Malashchuk, Brian R. Lajoie, Andrei N. Mardaryev, Michal R. Gdula, Andrey A. Sharov, Terumi Kohwi-Shigematsu, Vladimir A. Botchkarev, Michael Y. Fessing

**Affiliations:** 1 Centre for Skin Sciences, Faculty of Life Sciences, University of Bradford, Bradford, United Kingdom; 2 Program in Systems Biology, University of Massachusetts Medical School, Worcester, Massachusetts, United States of America; 3 Department of Biochemistry, University of Oxford, Oxford, United Kingdom; 4 Department of Dermatology, Boston University School of Medicine, Boston, Massachusetts, United States of America; 5 Department of Orofacial Sciences, School of Dentistry, University of California San Francisco, San Francisco, California, United States of America; Icahn School of Medicine at Mount Sinai, UNITED STATES

## Abstract

Mammalian genomes contain several dozens of large (>0.5 Mbp) lineage-specific gene loci harbouring functionally related genes. However, spatial chromatin folding, organization of the enhancer-promoter networks and their relevance to Topologically Associating Domains (TADs) in these loci remain poorly understood. TADs are principle units of the genome folding and represents the DNA regions within which DNA interacts more frequently and less frequently across the TAD boundary. Here, we used Chromatin Conformation Capture Carbon Copy (5C) technology to characterize spatial chromatin interaction network in the 3.1 Mb Epidermal Differentiation Complex (EDC) locus harbouring 61 functionally related genes that show lineage-specific activation during terminal keratinocyte differentiation in the epidermis. 5C data validated by 3D-FISH demonstrate that the EDC locus is organized into several TADs showing distinct lineage-specific chromatin interaction networks based on their transcription activity and the gene-rich or gene-poor status. Correlation of the 5C results with genome-wide studies for enhancer-specific histone modifications (H3K4me1 and H3K27ac) revealed that the majority of spatial chromatin interactions that involves the gene-rich TADs at the EDC locus in keratinocytes include both intra- and inter-TAD interaction networks, connecting gene promoters and enhancers. Compared to thymocytes in which the EDC locus is mostly transcriptionally inactive, these interactions were found to be keratinocyte-specific. In keratinocytes, the promoter-enhancer anchoring regions in the gene-rich transcriptionally active TADs are enriched for the binding of chromatin architectural proteins CTCF, Rad21 and chromatin remodeler Brg1. In contrast to gene-rich TADs, gene-poor TADs show preferential spatial contacts with each other, do not contain active enhancers and show decreased binding of CTCF, Rad21 and Brg1 in keratinocytes. Thus, spatial interactions between gene promoters and enhancers at the multi-TAD EDC locus in skin epithelial cells are cell type-specific and involve extensive contacts within TADs as well as between different gene-rich TADs, forming the framework for lineage-specific transcription.

## Author summary

Gene activity programmes in different cell types control development and homeostasis of multi-cellular organisms. Spatial genome organization controls gene activity by facilitating or restricting contacts between gene promoters and remote gene enhancers. Functionally related co-regulated genes are often located together in genomes loci. The spatial organization of very large co-regulated gene loci remains poorly understood. We analyse the spatial contact network in the Epidermal Differentiation Complex (EDC) locus that contains 61 co-regulated genes activated during epidermal differentiation in epidermal cells and thymocytes, where the locus is mostly inactive. Our analysis demonstrated that the gene-rich and gene-poor regions in the EDC are organized in separate Topologically Associating Domains (TADs). We further found that spatial contact in the EDC locus is mostly cell type specific. In keratinocytes such contacts connect gene promoters with gene enhancers both within and between gene-rich TADs. Chromatin architectural proteins CTCF and Rad21 together with chromatin remodeller Brg1 were often bound near the spatially contacting gene promoters and enhancers in keratinocytes. In contrast to gene-rich TADs, gene-poor TADs show preferential spatial contacts with each other, do not contain active enhancers and show decreased binding of CTCF, Rad21 and Brg1. These data illustrate how the chromatin networks required for lineage-specific transcription are organized in skin epithelial cells and demonstrate that spatial interactions involving gene promoters and enhancers at the EDC locus are not restricted by the TAD boundaries and involve, together with intra-TAD interactions, the extensive contacts between the different TADs.

## Introduction

Metazoan development requires the concerted specification of divergent lineages among a genetically homogenous cell population and the tightly controlled, coordinate genesis of cellular structural and functional diversity driven by proper spatial and temporal regulation of transcription. Genome topology in the nucleus plays an important role in regulation of gene transcription by facilitating or restricting spatial interactions between gene promoters and distal gene regulatory elements [1–7].

In the interphase nucleus, chromosomes occupy distinct positions called chromosome territories with some intermingling between the borders of the neighboring chromosomes [8]. Each chromosome is organized into Topologically Associating Domains (TADs), principal units of the chromatin folding that might be further divided into sub-TADs [9–11]. TADs range in size from several hundred Kb up to about 1.5 Mb in mice and humans. TADs are defined as chromatin domains with higher frequency of spatial contacts within the domains compared to the regions across TAD borders [9, 10, 12]. TAD borders are mostly conserved between the different cell types and mammalian species [9, 12, 13], although lineage-specific differences in the TAD borders have been described [9, 12].

The spatial chromatin contacts involve interactions between proximal gene promoters and distal gene regulatory regions, such as enhancers, silencers, insulators and locus control regions. These interactions vary substantially between different cell types and change during cell differentiation [6, 11, 12, 14]. The spatial interactions between gene promoters and enhancers mostly occur within TADs [15–17]. However, less frequent inter-TAD contacts occur between the transcriptionally active loci, and these often represent enhancer-promoter contacts that are largely cell-type specific [17–19]. The functional significance of the inter-TAD contacts remains to be further determined.

Spatial genome organization is controlled, at least in part, by a number of chromatin architectural proteins including CCCTC- binding factor (CTCF), Cohesin, condensin together with the Mediator co-activator complex [11, 20–22]. CTCF binding is often detected at the TAD borders, although most CTCF bound regions are found inside the TADs [9, 10, 12]. Cohesin is frequently, but not always, binds together with CTCF at the bases of chromatin loops [14, 20, 21, 23]. Cohesin controls spatial contacts between gene promoters and enhancers together with or independently of CTCF [11, 20, 22]. The Mediator complex is also frequently involved in the promoter-enhancer interactions together with cohesin [11, 22, 24].

Functionally-related and co-regulated genes frequently form conserved clusters or loci in the mammalian genomes, which size varies from several kilobases to several megabases [25, 26]. In mouse genome, there are several dozens of the large (more than 0.5 Mbp) gene loci, in which gene transcription is frequently regulated in a lineage-specific manner [27, 28]. Large lineage-specific gene loci are present on a vast majority of chromosomes and harbour the olfactory receptor family genes (chromosomes 2, 7, 9, 10, 14, 16, 17, 19), immunoglobulin kappa and heavy chain genes (chromosomes 6 and 12, respectively), keratinocyte-specific genes (chromosomes 3, 11, 15 and 16), as well as some other gene families [29]. The detailed chromatin conformation capture analysis of the several lineage-specific gene loci, including the Hox, beta–globin and protocadherin genes, revealed the importance of their proper spatial organization in executing lineage-specific gene expression programs by restricting the promoter-enhancer contacts to individual TADs [21, 30–32]. However, high resolution mapping of the chromatin interaction networks in the large lineage-specific loci and their relevance to the distinct TADs remain largely unexplored.

Epidermal Differentiation Complex (EDC) is a unique large locus in the mouse genome containing 61 functionally-related genes occupying 3.1 Mb domain in the gene-rich region of mouse chromosome 3 or 1.6 Mb domain on human chromosome 1 [33–35]. Central part of the EDC locus contains functionally-related genes involved in the control of epidermal differentiation and barrier acquisition, while two flanking EDC regions harbour the *S-100* family genes involved not only in epidermal differentiation, but also playing various functions in other tissues [33–35]. In mouse genome, the central part of the EDC is separated from its 5’- (centromere proximal) domain by a gene desert, while another gene-poor domain separates the 3’-flank of the EDC from the neighbouring gene-rich domain on chromosome 3 [33–35].

During epidermal morphogenesis and transition of the single-layered surface epithelium (E11.5) to stratified epidermis (E16.5), higher-order chromatin folding of the EDC harbouring region on the mouse chromosome 3 show remarkable plasticity resulting in relocation of the EDC from the nuclear periphery towards nuclear interior [36]. These changes are associated with remodelling of chromatin compaction in the central EDC domain and increased transcription of many EDC genes involved in terminal keratinocyte differentiation [29]. Developmentally-regulated higher-order chromatin remodelling of the EDC locus in keratinocytes is orchestrated by the epidermal master transcription regulator p63, which directly regulates expression of the ATP-dependent remodeller Brg1 and genome organizer Satb1 in the epidermal progenitor cells [29, 36]. In turn, Brg1 controls the developmentally-regulated relocation of the EDC towards the nuclear interior, while Satb1 promotes establishing the proper level of chromatin compaction in the central EDC domain required to maintain or balance gene transcription in the locus in terminally differentiating keratinocytes [29, 36].

In spite of the essential role of the EDC locus in epidermal differentiation and barrier acquisition, surprisingly little is known about the distal gene regulatory elements in this region and their interactions with the target gene promoters. Several non-coding regulatory elements showing the enhancer or silencer activities were identified in this locus based on the non-coding sequence homology in mammalian species [33]. 3C studies demonstrated the long-range spatial contacts between a conserved AP-1 dependent gene enhancer with the selected gene promoters in this locus in cultured epidermal keratinocytes [37]. However, the comprehensive pattern of spatial chromatin contacts in the EDC locus, including its organization into distinct TADs, promoter-enhancer regulatory network and the factors involved in its establishment and maintenance remain unexplored.

Here, we map the spatial chromatin contacts at the EDC and neighbouring genome region in murine basal epidermal keratinocytes and thymocytes (used as a control in which the keratinocyte-specific genes at the EDC are inactive), at high resolution employing the Chromosome Conformation Capture Carbon Copy (5C technology). We demonstrate that in keratinocytes, the EDC locus is organized into several gene-rich and gene-poor TADs and forms lineage-specific spatial contact networks. Furthermore, in keratinocytes, in addition to the intra-TAD contacts, a substantial number of keratinocyte-specific spatial interactions connecting putative gene enhancers with promoters were detected between different gene-rich TADs. We also show enrichment for binding of CTCF, Rad21, and ATP-dependent chromatin remodeller Brg1 in the spatial enhancer-promoter contacts within and between gene-rich TADs, suggesting their roles in the establishment of the unique spatial chromatin organization and control of gene expression in the large multi-TAD EDC locus in skin epithelial cells.

## Results

### Large lineage-specific gene loci constitute several TADs in the genome, while smaller-sized loci are predominantly located within individual TADs

**Fig 1:**
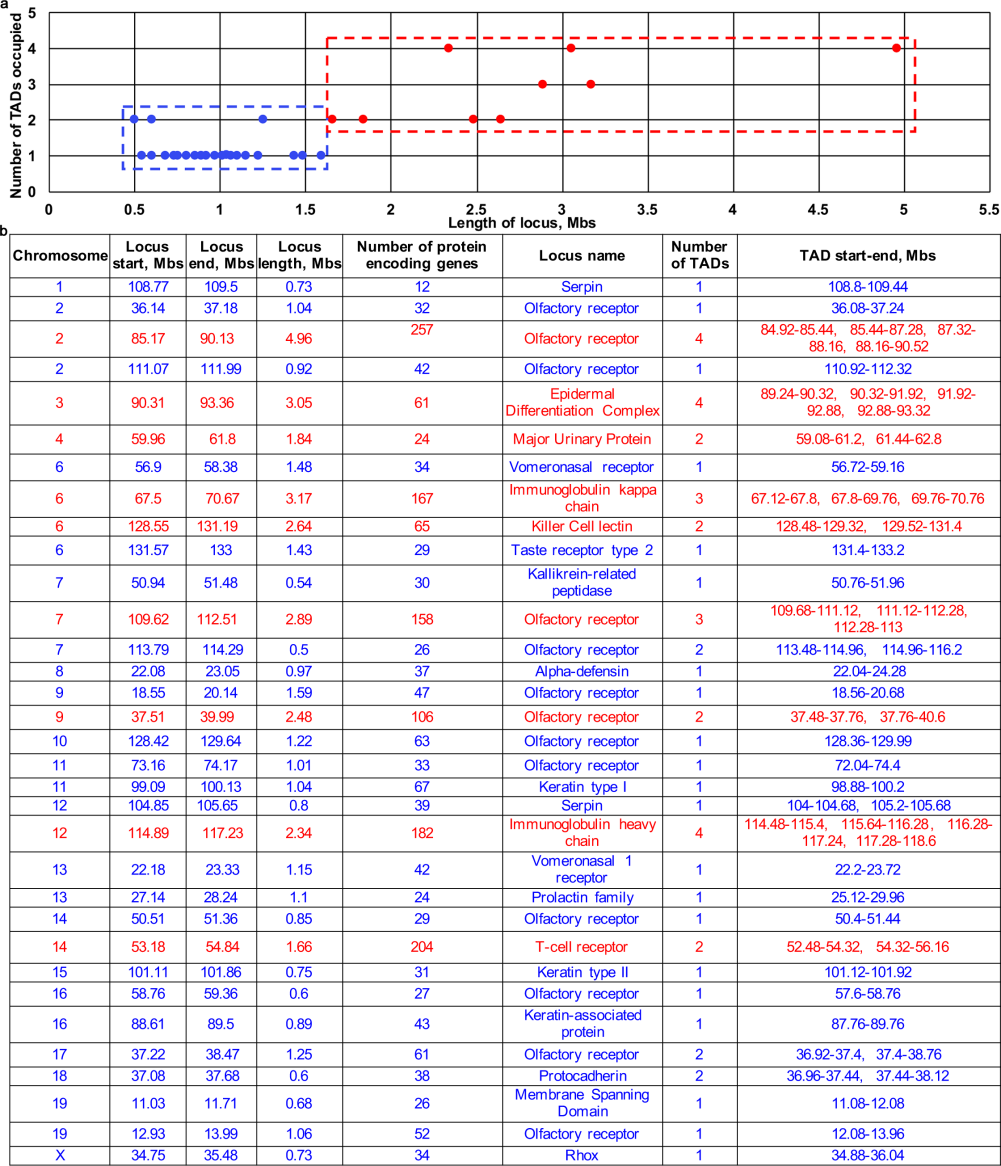
TAD organization of the large (>0.5 Mb) lineage-specific co-regulated gene loci in the mouse genome. **(a)** Number of TADs occupied by the lineage-specific co-regulated gene loci in the mouse genome in the Embryonic Stem Cells (ESCs) depending of the locus size (based on the Hi-C data published in [9]). **(b)** Genome positions of the lineage-specific co-regulated gene loci in mouse genome and the position of the TAD borders occupied by these loci in the ESCs (mouse genome assembly mmu9), based on the Hi-C data published in [9]).

In the mouse genome, there are 33 large (more than 0.5 Mbp) lineage-specific gene loci containing at least 10 functionally related genes [27–29] (**Fig 1**). Large lineage-specific gene loci are present on a vast majority of chromosomes and harbour the olfactory receptor family genes (chromosomes 2, 7, 9, 10, 14, 16, 17, 19), immunoglobulin kappa and heavy chain genes (chromosomes 6 and 12, respectively), keratinocyte-specific genes (chromosomes 3, 11, 15 and 16), as well as some other gene families (**Fig 1A and 1B**).

To correlate the genomic location of such loci to the distinct TADs genome-wide, we used the TAD maps generated using Hi-C technology for mouse embryonic stem cells [9]. Interestingly, this analysis revealed that among 24 lineage-specific loci occupying between 0.5–1.6 Mbs in the genome, 21 loci were localized within single TADs on the corresponding chromosomes, while only 3 loci were spread between two neighbouring TADs (**Fig 1A and 1B**). Epithelial-specific gene loci, such as Keratin type I and type II [KtyI/II] loci, Keratin-associated protein [KAP] locus having size between 0.75–1 Mbs, were localized within individual TADs on mouse chromosomes 11, 15 and 16, respectively (**Fig 1B**).

However, 100% lineage-specific loci of larger size (>1.6 Mb) including Epidermal Differentiation Complex [EDC] locus [34] were occupying several (from two to four) TADs on the corresponding chromosomes (**Fig 1A and 1B**). Because TADs were defined as genomic regions with a higher frequency of spatial contacts within the domains compared to inter-domain interactions [9, 10, 12], these data raised the questions whether large multi-TAD lineage-specific gene loci display any unique features in the chromatin interaction patterns between functionally related genes localized in different TADs and how such interactions are regulated.

### EDC locus in keratinocytes is organized into four Topologically Associating Domains with distinct compartmentalization patterns based on their gene-rich or gene-poor status

**Fig 2:**
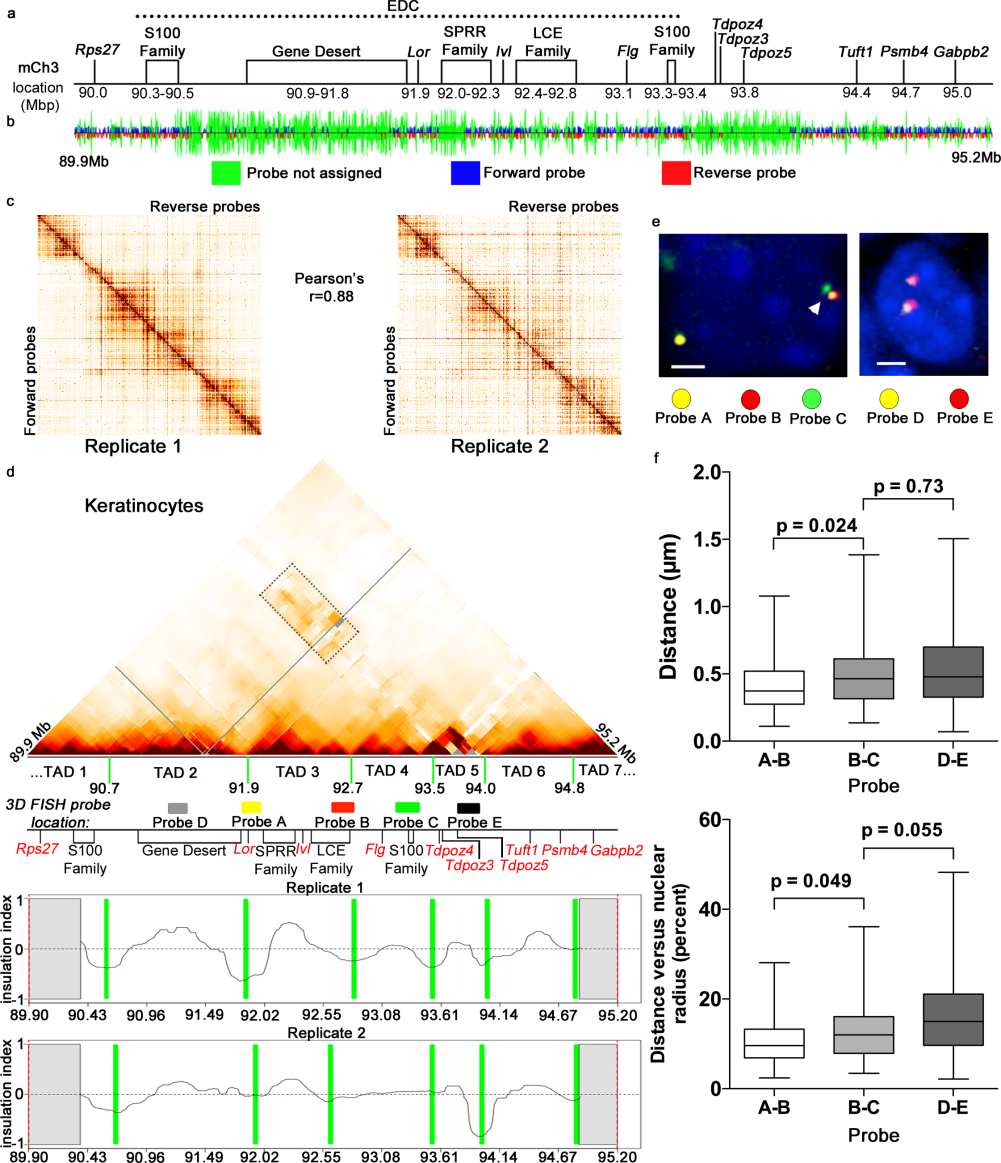
Gene-rich and gene-poor regions are organized into distinct TADs at the EDC locus in keratinocytes. **(a)** Schematic structure of the 5.3 Mb genomic region containing the EDC locus on mouse chromosome 3 analysed using 5C technology in this manuscript (mm9/chr.3:89,900,000–95,200,000). **(b)** Alternating 5C probe design for the unique HindIII sites in the interrogated genomic regions. The position of the restriction sites interrogated by the forward primers are shown in blue, interrogated by the reverse primers are shown in red and the site for which the primers could not be designed are shown in green. **(c)** Heatmaps representing raw 5C data for both KC replicates. Reverse probes are plotted as columns and the forward probes as rows. Pearson’s correlation coefficient is also shown. **(d)** Heatmap representing the 5C data after the normalization and binning (bin size 150 kb, step size 15kb) in KCs. The position of TAD border midpoints (average for the midpoints calculated based on the insulation index analysis in two replicates independently) are identified by green lines under the heatmaps. Note the high frequency of the spatial contacts between the gene-poor TADs 2 and 5 (indicated by dashed rectangle on the heat map). The position of the regions covered by the BAC fish probes used in these studies, schematic map of the studied locus and insulation indexes profiles for two 5C library replicates are also shown. **(e)** Multi-colour 3D FISH analysis with BAC probes A (located at the 5’ border of TAD3, B (located at the 3’ border of TAD4) and C (located within TAD4) (left)**,** or with BAC probe D (located within gene-poor TAD2) and E (located within gene-poor TAD5) (right) in basal epidermal keratinocytes. Representative single optical sections are shown. Scale bars are 2μm. **(f)** Box plots showing median, 25% quartile, 75% quartile with whiskers indicating maximum and minimum for spatial distances between the centres of the regions covered by probes A and B, probes B and C, as well as probes D and E before (in μm) and after normalization to the average nuclear radius (in % of average nuclear radius) in basal epidermal keratinocytes *in situ*. The distances between the centres of the regions covered by the probes A and B (located within TAD3) are significantly shorter than the distances between loci covered by the probes B and C (located within TAD4). The indicated p-values for pair-wise comparison are calculated using Mann-Whitney U-test, n = 60 alleles for each interrogated locus. The distances between the centres of the regions covered by the probes D and E (located in the gene poor TADs 2 and 5 respectively) are similar to the much closer regions covered by the probes B and C (located in the adjacent gene-rich TAD3 and TAD4).

To address this question, we focused on the EDC locus occupying ~3.1 Mb in one of the most gene-dense regions of mouse chromosome 3 [29]. Its central domain consists of the co-regulated genes involved in the control of terminal keratinocyte differentiation and epidermal barrier acquisition, including *Loricrin (Lor)*, the *Small proline-rich (Sprr)* gene family, *Involucrin (Ivl)*, *Late cornified envelope (Lce)* gene family and *Fillagrin*-like (*Flg*-like) gene family [33, 36, 38] **(Fig 2A)**. The *S100* family genes flank the 5’- and 3’-ends of the EDC [38] **(Fig 2A).** In addition to the gene-rich domains, the EDC locus in mice contains a gene-poor region (“desert”) separating the part of *S100* family genes at 5’ of the EDC and the *Lor* gene at central EDC domain, while another gene-poor domain separates the 3’-flank of the EDC from the neighbouring gene-rich domain on chromosome 3 **(Fig 2A, S1 Table)**.

To study the potential connection between gene activity and spatial chromatin folding in the EDC locus at higher resolution, we correlated gene expression determined by microarray profiling with data obtained with Chromosome Conformation Capture Carbon Copy (5C) technology in freshly plated neonatal epidermal keratinocytes. Consistently with the data demonstrating specific roles for many genes that constitute the central EDC domain in the control of epidermal barrier formation [39–42], microarray showed that in epidermal keratinocytes most of these genes were expressed at much higher levels compared to thymocytes, used as the control in which those genes were inactive **(S1A Fig, S1 Table)**.

5C is well-suited for analyses of the spatial genome folding, as it allows the simultaneous detection of the spatial chromatin looping contacts and identification of TADs [17, 43]. For the 5C analysis, 381 forward and 382 reverse 5C probes were designed in an alternating scheme using the tools from my5C software suite [44] to interrogate HindIII sites with the unique anchoring regions at the EDC and its flanking regions (mm9, chromosome 3: 89.9–95.2 Mbp) **(Fig 2B, S2 Table).** The designed probe pool interrogated 145,542 potential pair-wise spatial chromatin contacts within this 5.3 Mbp genomic region. Two biological 5C library replicates were generated and analyzed for each cell type.

Consistently with previous studies using 5C and Hi-C technologies [9–11, 45], the raw 5C data for both replicates (shown as heatmaps with all reverse probes plotted as columns and forward probes as rows (**Fig 2C, S1B Fig**), clearly demonstrated that neighbouring chromatin regions interact to each other frequently, creating a black “diagonal” in the middle part of the heatmaps. Raw 5C data showed high similarity between the biological replicates for both cell lineages **(Fig 2C, S1B Fig)**, and the raw 5C counts highly correlated between the replicates (for keratinocyte libraries—Pearson correlation coefficient 0.88; for thymocyte libraries—Pearson correlation coefficient 0.94), indicating a high quality of our 5C data (**Fig 2C, S1B Fig**). However, the correlations between the keratinocyte and thymocyte libraries were much lower (Pearson correlation coefficient 0.61), indicating lineage-specific differences in folding of the locus between both cell types.

Correction of the 5C data for non-biological biases associated with this technology was performed as described previously [17, 43] (see Materials and Methods for details) **(S2–S5 Figs)**. The corrected data were binned (bin size 150kb with the step size of 15kb) to account for the differences in the 5C probe coverage in the different parts of the 5.3 Mbp genomic region **(Fig 2D, S2E Fig, S3E Fig, S4E Fig, S5E Fig and S6A Fig).** The heatmaps representing 5C data clearly showed several consecutive chromatin regions with high spatial self-associations (visible as darker “triangles” above a black “diagonal”) corresponding to the distinct TADs in keratinocytes and thymocytes **(Fig 2D, S6A Fig)** [9, 10, 17]. To define the positions of the TAD boundaries, we performed the insulation index analysis on each replicate data set separately, as described elsewhere [17, 46] (see Materials and Methods for details) **(Fig 2D, S6A Fig, S3 Table).** This analysis identified the boundaries separating TADs in the 5.3 Mbp region in keratinocytes and thymocytes **(Fig 2D, S3 Table).** The accuracy of the TAD boundary calculations performed by comparing the determined boundary midpoint positions between the replicates **(S3 Table)** indicated that TAD boundary positions were determined with the accuracy of about +/- 100 kb.

5C data revealed that 5.3 Mbp chromatin domain containing EDC locus on mouse chromosome 3 is organized into seven distinct TADs **(Fig 2D, S3 Table).** We calculated density of the protein-coding genes in these TADs and correlated the results to the average gene density in the mouse genome (75 kb per gene). Based on these analyses, we defined the gene-rich (21–36 Kb per gene) and gene-poor (166–400 Kb per gene) TADs in the EDC locus (**S1 and S3 Tables**). 5’-flanking region of the EDC locus containing the *S100* family genes constituted the part of the gene-rich TAD1, which also harbours neighbouring non-EDC genes including the house-keeping *Rps27* gene. Gene-poor domain of the EDC separating S100 family genes from its central domain constituted the TAD2. In turn, the central EDC domain and its 3’ flank region were organized into two distinct TADs: *Lor* gene was located at the boundary between TAD2 and TAD3, which also contained the *Ivl* gene, *Sprr* gene family and the major part of the *Lce* gene family, while TAD4 encompassing the remaining part of the *Lce* gene family, *Flg*-like gene family, 3’-flanking part of *S100* gene family, and the part of *Tdpoz* gene family. The chromatin domain located further outside of the 3’-end of the EDC locus was organized into gene-poor TAD5 containing the remaining part of the *Tdpoz* gene family, as well as into gene-rich TAD6 and part of the TAD7, respectively **(Fig 2D, S3 Table)**.

These data were quite consistent with the Hi-C data obtained from mouse embryonic stem cells [9] (**Fig 1B, S1 Table**), as well as with 5C data obtained from thymocytes. In thymocytes, the border between TAD T1 and TAD T2 (90.8 Mb), as well as between TAD T2 and TAD T3 (92.1 Mb) were only slightly shifted compared to keratinocytes (90.7 Mb and 90.9 Mb, respectively) **(S6A and S3 Tables)**. Similar to keratinocytes, the central EDC domain in thymocytes was organized into TAD3 (92.1–92.7 Mb) and TAD4 (92.7–93.9 Mb) (**S6A Fig, S3 Table**). However, TAD T4 and TAD5 in thymocytes did not show clear separation and the border between them was rather softened (**Fig 2D, S6A Fig, S3 Table**). The borders between the TAD 4/5 and TAD 6 (93.9 Mb), as well as between TAD6 and TAD7 (94.8 Mb) in thymocytes were quite similar compared to the corresponding borders in keratinocytes **(Fig 2D, S6A Fig, S3 Table)**.

Importantly, the TAD borders were weaker in thymocytes versus keratinocytes, while the frequency of the spatial inter-chromatin contacts both within and between different TADs in keratinocytes was substantially higher in comparison to thymocytes **(Fig 2D, S6A Fig).** Interestingly, we observed high frequency of the spatial chromatin contacts between the gene-poor TAD2 and TAD5, flanking the gene-rich TAD3 and TAD4 in keratinocytes, while such interactions were not seen in thymocytes **(Fig 2D, S6A Fig)**. Such high frequency of contacts was not observed on the heat map between TAD1 and TAD3, separated by the gene-poor TAD2 in keratinocytes **(Fig 2D)**. These data suggested that the gene-poor TADs at the 5.3 Mbp chromatin domain on mouse chromosome 3 appears to be segregated into transcriptionally-inactive compartment spatially separated from the transcriptionally active gene-rich TADs in keratinocytes, which is quite consistent with the model proposing the existence of the compartments A and B topologically separated in the nucleus based on the differences in their transcription activity [9, 45]. Importantly, such separation was not observed for the transcriptionally inactive gene-rich and gene-poor TADs in thymocytes **(S6A Fig)**, presumably incorporated into compartment B in these cells.

### 3D-FISH analysis confirms the lineage-specific topological organization of the EDC locus and spatial segregation of the gene-rich and gene-poor TADs in keratinocytes

To validate the 5C data, we performed 3D-FISH analysis of the distances between loci located in the distinct EDC domains in the freshly plated primary epidermal keratinocytes and thymocytes, as well as in cryo-sections of P0.5 mouse skin *in situ*. First, we checked if the central part of the EDC is indeed organized into two adjacent gene-rich TAD3 and TAD4 in both cell types **(Fig 2D, S6A Fig)**. For the 3D FISH analysis, we have chosen the BAC probes depicting the regions within the TAD3 near its 5’ and 3’ borders (probes A and B, respectively), or located within the adjacent TAD4 (probe C) (**S4 Table, Fig 2D–2F, S6B Fig**). We expected that the spatial distances between the regions located within the same TAD should be shorter in comparison to the distances between the regions located in the different TADs, when the similar linear genomic distances separate such regions [9, 10].

Indeed, 3D-FISH analyses demonstrated that despite the fact that the genomic distances between the centers of the regions covered by the probes A and B located within the same TAD3 were slightly longer (716,849 bp) compared to the distances between the regions covered by the probes B and C (638,779 bp) located in the TAD3 and TAD4, respectively (**S4 Table**), spatial distances between the centers of the 3D FISH signals generated by the probes A and B were significantly shorter compared to the distances between the probes B and C in all cell populations **(Fig 2D–2F, S6A Fig, S5 Table)**. Thus, this analysis confirmed the folding of the EDC central domain into two separate TADs both in keratinocytes and thymocytes.

Importantly, 3D-FISH data also showed that the spatial distances between the 3D-signals were rather similar in basal epidermal keratinocytes *in situ* and in the freshly isolated epidermal keratinocytes **(Fig 2F, S6B Fig, S5 Table)**, thus confirming that the cell isolation procedure for 5C does not significantly alter the spatial organization of the EDC locus. However, 3D FISH analysis also revealed that the distances between the probes A—B and B—C were significantly larger in thymocytes compared to keratinocytes (p<0.0001, Mann-Whitney U-test) (**S6B Fig, S5 Table**), demonstrating that chromatin in the transcriptionally inactive domains of the EDC locus in thymocytes is less condensed and likely to be more randomly folded compared to the active locus in keratinocytes.

Next, we checked whether gene-poor TAD2 and TAD5 are indeed located closely to each other in keratinocytes, as this has been suggested by the 5C data (**Fig 2D**). We performed the 3D-FISH analysis of the basal epidermal KCs *in situ* using the probes covering the centre of the TAD2 (probe D) and TAD5 (probe E) **(Fig 2D, S4 Table)**. TAD2 and TAD5 are separated from each other in the genome by the gene-rich TAD3 and TAD4 (**Fig 2D**). 3D-FISH data showed that despite the genomic distances between the regions covered by the probes D and E were much longer (2,610,522 bp) compared to the distances between the probes B and C that depict TAD3 and TAD4 (638,779 bp), the spatial distances between the probes D and E, as well as between the probes B and C, were quite similar **(Fig 2F, S5 Table)**. These data demonstrated close association of the gene-poor TAD2 and TAD5 in keratinocytes, thus demonstrating the consistence with the 5C results.

Thus, 3D-FISH analyses confirmed the organization of the central and 3’-flanking regions of the EDC into two separate gene-rich TADs in both cell lineages, as well as the compartmentalization of the gene-poor TAD2 and TAD5 in keratinocytes. Furthermore, this analysis also confirmed the less condensed and potentially more randomly spatially organized the transcriptionally inactive locus in thymocytes in comparison to the active locus in keratinocytes. The concordance between the 5C and 3D-FISH data, as well as between 3D-FISH data obtained from isolated keratinocytes and basal epidermal keratinocytes *in situ* suggested that the gene-rich and gene-poor TADs in the EDC locus and its neighbouring regions indeed form a unique and relatively stable spatial composition that might serve as a platform for the control of lineage-specific transcription.

### 5C chromatin contact network at the EDC locus in keratinocytes includes distinct patterns of interactions between different gene-rich and gene-poor TADs

**Fig 3:**
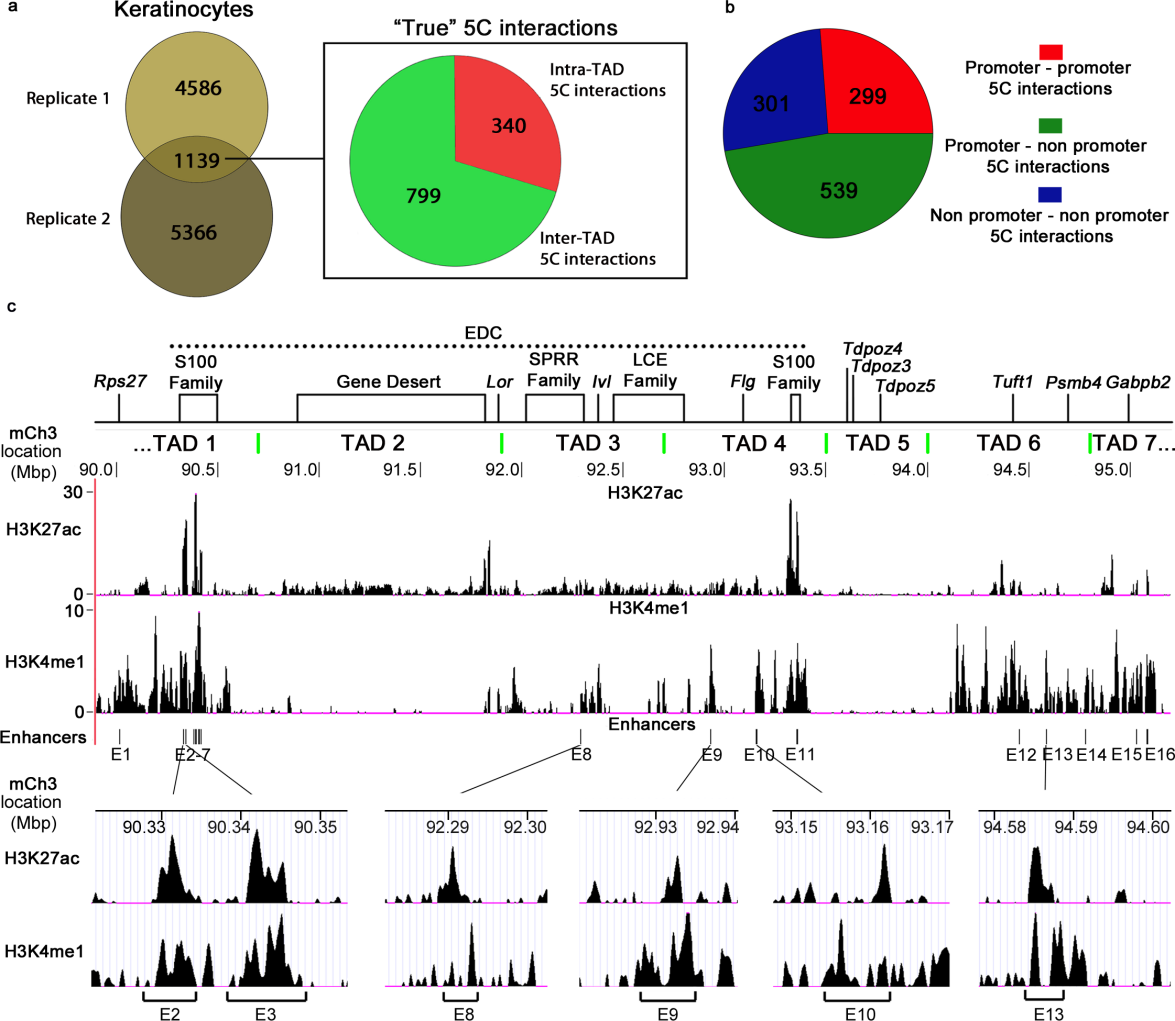
5C looping interactions at the EDC locus involve gene promoters and enhancers in keratinocytes. **(a)** Vent diagram indicating the overlap of the significant 5C interactions (q<0.05) between the 5C library replicates and pie chart showing the number of all “true” intra-TAD (red) and inter-TAD (green) 5C interactions in KCs. **(b)** Pie-chart indicating number of 5C interactions connecting two regions anchoring transcription start sites (TSSs) within 5kb (promoter-promoter interactions); one contacting region anchoring a TSS within 5kb and the other contacting region not anchoring a TSS within 5 kb (promoter-non promoter interactions); and both contacting regions not anchoring TSSs within 5kb (non-promoter–non promoter interactions). **(c)** Genome browser images of the normalized ChIP-seq signals for H3K4me1 and H3K27ac enrichment as well as the position of the putative gene enhancers at the EDC containing locus in KCs aligned to the schematic locus map. Genome browser images of the normalized ChIP-seq signals for several enhancer regions at small scale are provided as examples. See Materials and Methods section for details of ChIP-seq peak calling and pursing the putative enhancers. The TAD border midpoints are indicated by the green lines.

To further characterise the spatial chromatin interaction network at the EDC locus and distinguish “true” chromatin interactions in the EDC locus from the random background interactions, we used an approach described previously [17, 43], which is based on the establishment of the background baseline defining the expected frequency of the random chromatin contacts normalized to the genomic distances separating the interacting fragments. This approach allowed identifying interactions reproducible in both 5C replicates with significantly higher interaction frequency compared to the background: 1139 “true” interactions in keratinocytes and 1033 interactions in thymocytes; q-value<0.05; (**Fig 3A, S6 Table, S6C Fig, S7 Table).** The reproducibility of the called 5C interactions between both replicates was similar to the previously published 5C datasets [11, 17, 43]. To compare the common and cell-type specific 5C interactions between keratinocytes and thymocytes, we also identified a subset of the interactions that were interrogated in all four 5C libraries after the 5C dataset normalization. This approach revealed 338 keratinocyte-specific 5C interactions, 747 thymocyte-specific interactions, while only 136 interactions were common between both cell types **(S6D Fig, S8–S10 Tables).** Thus identification of the “true” 5C interactions in keratinocytes and thymocytes further demonstrated that spatial organization of the EDC locus is largely lineage-specific.

To further characterize the patterns of the 5C interactions in the transcriptionally active EDC locus in keratinocytes and to check if there are differences in the frequency of the “true” 5C spatial contacts within and between different TADs, we used the 5C contact sets reproducible in both keratinocyte libraries **(S3A Fig, S6 Table).** Interestingly, we identified substantially more 5C interactions between different TADs (799 or 70.15%), than within the individual TADs (340 or 29.85%) **(Fig 3A).** Analyses of the 5C interactions between different TADs revealed that gene–rich TAD3 and TAD4 harbouring the majority of genes activated during terminal keratinocyte differentiation interact equally extensively with the gene-rich TAD1 and gene-poor TAD2 (**S6E Fig**). However, TAD1 harbouring a part of the S100 family genes showed a markedly decreased number of interactions with neighbouring gene-poor TAD2 compared to more distantly located TAD3 and TAD4 (**S6E Fig**). Remarkably, gene-poor TAD5 showed preferential interactions with gene-poor TAD2, which, in turn, interacted quite extensively with the gene-rich TAD3 and TAD4 (**S6E Fig)**. Gene-rich TAD6 that does not contain keratinocyte-specific genes also interacted quite extensively with TAD1, TAD2, TAD3 and TAD4, while showed only very limited number of interactions with neighbouring gene-poor TAD5 (**S6E Fig**).

Next, we checked the frequency of the inter-TAD and intra-TAD 5C interactions at the EDC locus as a function of the genomic distances separating contacting fragments in keratinocytes. Surprisingly, we found that the frequency of all detected spatial contacts within the TADs were generally only slightly higher in comparison to the contacts between the TADs (**S6F Fig**) Such extensive chromatin interaction network between different neighbouring gene-rich TADs harbouring the lineage-specific genes, as well as lineage-specific folding of the EDC locus suggests the functional relevance of these contacts for coordination of the gene expression in keratinocytes during execution of epidermal differentiation program.

### Long-range chromatin contact network within and between gene-rich TADs at the EDC locus in keratinocytes involves gene promoters and enhancers

5C analysis demonstrated that majority of all 1139 “true” 5C interactions in keratinocytes (47.3%) involve the contacts between the gene promoters and non-promoter chromatin domains, while considerably lower number of interactions were involving either two promoters (26.3%) or two non-promoter chromatin domains (26.4%), respectively **(Fig 3B)**. Thus, vast majority of the 5C contacts (73.6%) involve the non-promoter elements (possibly including gene enhancers) at the EDC locus in keratinocytes.

To further characterize the 5C interactions between gene promoters and enhancers, we identified putative gene enhancers in the EDC locus and its neighbouring regions by performing ChIP-seq analysis for enhancer-specific histone modifications with anti-H3K4me1 and anti-H3K27ac antibodies on the freshly isolated FACS sorted basal (Integrin 6 alpha high, Sca1 high) mouse epidermal keratinocytes. ChIP-seq analyses revealed 16 regions in the EDC locus and its neighbouring regions with the high levels of both H3K4me1 and H3K27ac modifications, serving as the signatures of active enhancers [3, 47, 48] **(Fig 3C, S11 Table).**

Interestingly, the putative active enhancers were identified exclusively in the gene-rich TADs: TAD1 (E1-E7), TAD3 (E8), TAD4 (E9-E11), TAD6 (E12-E14) and TAD7 (E15-E16) **(Fig 3C)**. Among these enhancers, two groups of closely located enhancers (within less than 10 kb distance from end to end for each enhancer: E2/E3 and E4-E7) formed two clusters (potential super-enhancers) within the TAD1, while the enhancers within other TADs were quite distantly located from each other and did not show clustering (serving probably as typical enhancers) **(Fig 3C, S11 Table)**. Moreover, lack of any enhancers was seen in the gene-poor TAD2 and TAD5.

**Fig 4:**
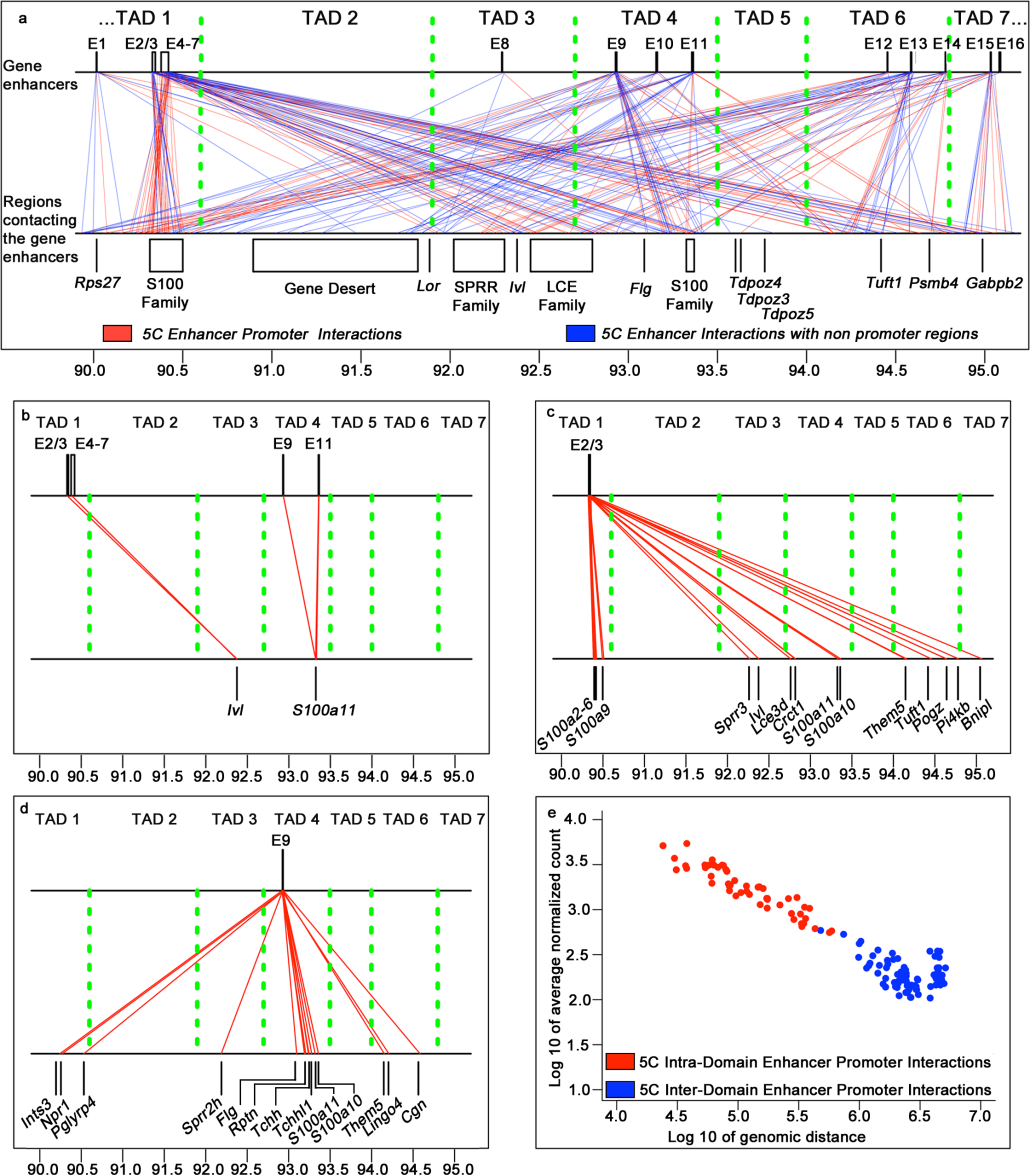
Spatial interaction networks between gene enhancers and promoters at the EDC locus in keratinocytes. **(a)** 5C looping interactions between gene enhancers (top line) and promoters (bottom line) (in red) and gene enhancers with regions not containing gene promoters (bottom line) (in bleu), TAD border midpoints are indicated by the vertical green lines. Schematic organization of the EDC locus is also shown. **(b)** 5C looping interactions involving *Ivl* and *S100a11* gene promoters (bottom line) with their enhancers (top line), TAD border midpoints are indicated by the vertical green lines. **(c)** 5C looping interactions between the enhancer cluster E2/E3 (top line) and its putative target gene promoters (bottom line), TAD border midpoints are indicated by the vertical green lines. **(d)** 5C looping interactions between the enhancer E9 (top line) and its putative target gene promoters. **(e)** Scaling plot showing the normalized average counts versus genomic distances for the 5C looping interaction between gene promoters and enhancers within the TADs (red), and between the TADs (blue).

To identify spatial interaction network between the enhancers and gene promoters, we assigned the 5C interactions involving the restriction fragments within 10 kb of each enhancer to either corresponding individual enhancers (E1, E8-E16) or to the clusters of closely located enhancers (E2/E3 and E4-E7). We found that 22% (252 out of 1139) of all 5C looping interactions at the EDC were anchored to the fragments bearing the gene enhancers **(S12 Table).** Then we assigned the closest gene transcription start sites (TSSs) located not further than 10 kb away from the restriction fragments anchoring the 5C interactions on the opposite side of the enhancers **(Fig 4A, S13 Table).** Our analysis revealed that about 52% (144 out of 273) of the 5C interactions involving gene enhancers were the interactions between gene enhancers and promoters, consistently with the data showing the involvement of the gene enhancers in long-range spatial contacts with the target promoters [43, 49, 50].

All enhancer-bearing regions, except the one for E16, were engaged in multiple spatial chromatin interactions with the regions anchoring gene promoters, revealing the potential enhancer-promoter regulatory network **(Fig 4A, S12 Table).** All enhancers or enhancer clusters except E8 were involved in the long-range contacts with multiple gene promoters, consistently with data obtained from other cell types [16, 43, 51] **(Fig 4A, S13 Table).** In turn, some gene promoters in the EDC locus were involved in the long-range spatial contacts with several enhancers. For instance, *Ivl* gene was involved in contacts with the enhancer clusters E2/E3 and E4-E7, while the *S100a11* gene was interacting with enhancers E9 and E11 **(Fig 4B, S13 Table)**, consistently with observations that gene promoters might interacts with several enhancers [17, 43, 49].

Enhancers were frequently involved in the spatial interactions not only with the gene promoters located in the same TADs, but also with the gene promoters located in the different TADs. For instance, in addition to the interactions with multiple gene promoters in the TAD1, a cluster of the enhancers E2/E3 (located in TAD1) were interacting with the regions containing *Sprr3 and Ivl* gene promoters in the TAD3, the *Crct1*, *Lce3d*, *S100a10* and *S100a11* gene promoters in the TAD4, the *Pi4kb*, *Pogz*, *Them5* and *Tuft1* gene promoters in TAD6, as well as with the *Bnipl* gene promoter in the TAD7 **(Fig 4C, S14 Table)**. Enhancer E9, located in the TAD4, spatially contacted the promoter regions of *Flg*, *Rptn*, *S100a10*, *S100a11*, *Tchh* and *Tchhl1* genes in the same TAD, as well as to the promoter regions of *Ints3*, *Npr1* and *Pglyrp4* genes in the TAD1, the promoter of *Sprr2h* gene in the TAD3, and promoters of *Cgn*, *Lingo4* and *Them5* genes in the TAD6 **(Fig 4D, S14 Table).** These data were quite intriguing, as many recent studies demonstrated that the contacts between promoters and enhancers are mostly constrained by the same TADs [15–17].

Interestingly, we also found a relatively low number of interactions between the enhancers located in gene-rich TADs with distinct chromatin domains located in gene-poor TAD2 and TAD5 **(Fig 4A, S12 Table).** The vast majority of such interactions involved distal elements not associated with any gene promoters in the TAD2 and TAD5, although interaction between the *Tdpoz3* gene promoter (TAD5) and the E14 enhancer located in TAD6 was also seen **(S14 Table).**

Next, we compared the frequency of promoter-enhancer contacts between and within TADs as a function of genomic distances separating interacting regions [17, 52]. We found that all promoter-enhancer spatial interactions within the TADs were connecting the regions separated by genomic distances of up to 0.6 Mb, while the inter-TAD interactions were much longer connecting the regions separated from each other by 0.5 Mb-5.1 Mb distances **(Fig 4E)**. As expected, the frequencies of short-range intra-domain contacts were higher compared to the long-range inter-TAD contacts. However, several inter- and intra- domain contacts found between the promoters and enhancers separated by similar genomic distances had comparable frequencies **(Fig 4E).** Thus, our data revealed the organization of the enhancer-promoter network in the EDC locus with a high frequency of short-range contacts within gene-rich TADs and less frequent, but extensive long-range promoter-enhancer interactions between gene-rich TADs, while gene-poor TADs were lacking of any enhancers.

### Chromatin architectural proteins CTCF and Rad21, and ATP-dependent chromatin remodeler Brg1 are enriched in spatial interactome connecting gene enhancers and promoters within and between gene-rich TADs in keratinocytes

To gain further insights about the proteins that could be potentially involved in the control of higher-order chromatin folding and promoter-enhancer interactions at the EDC locus in keratinocytes, we correlated the 5C interaction data with the ChIP-seq data for the binding of the chromatin architectural proteins CTCF and cohesin subunit Rad21, known to control the higher-order chromatin folding in all studied cell types [4, 53]. We also correlated 5C data with ChIP-seq data for the binding of ATP-dependent chromatin remodeler Brg1/Smarca4, known to regulate nuclear positioning of the EDC locus in keratinocytes during epidermal development [36].

**Fig 5:**
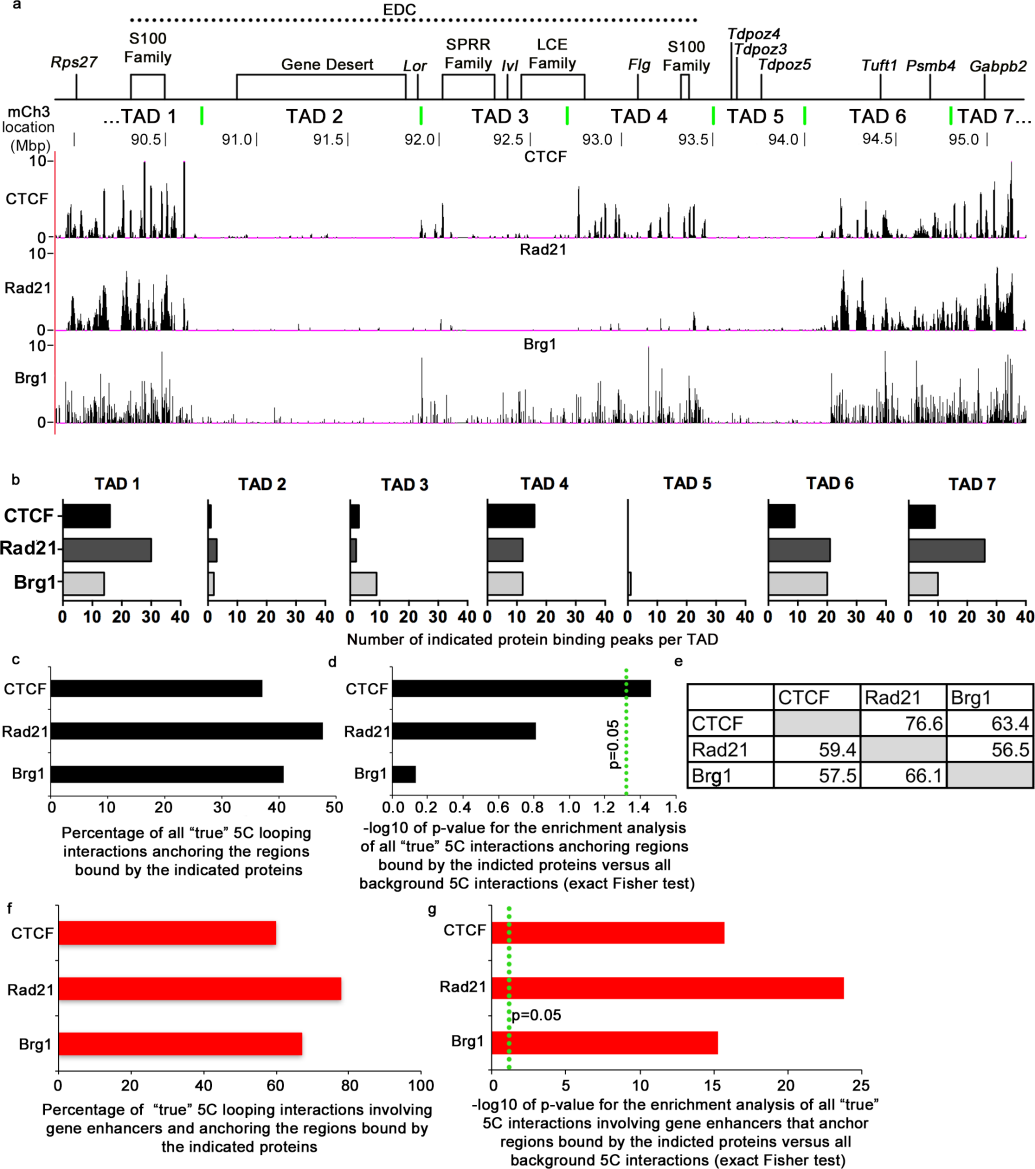
Chromatin architectural proteins CTCF, Rad21 and ATP-dependent chromatin remodeller Brg1 are enriched in the regions involved in the promoter-enhancer spatial interactions at the EDC locus. **(a)** Schematic map of the EDC containing locus and genome browser view of the normalized ChIP-seq signals for the indicated proteins. The TAD border midpoints are indicated by the green vertical lines. **(b)** Number of the called ChIP-Seq peaks for the indicated proteins in the individual TADs **(c)** Percentage of all “true” 5C looping interactions anchoring the regions bound by the indicated proteins. **(d)** Results of the enrichment analysis of 5C interactions anchoring the regions bound by the indicated proteins in comparison to all the background interactions at the EDC containing locus.–log10 of the p-values are shown (exact Fisher test). **(e)** Percentage of the significant 5C interactions anchored to the regions bound by the proteins indicated in the left column that are also anchored to the regions bound by the proteins indicated in the top row. **(f)** Percentage of 5C interaction involving gene enhancers that are anchor the regions bound by the indicated proteins.**(g)** Results of the enrichment analysis for the 5C interactions involving gene enhancers that are anchored to the regions bound by the indicated proteins in comparison to all background interactions in the EDC locus.–log10 of the p-values are shown (exact Fisher test).

We found a heterogeneous distribution in the binding patterns for these proteins at the EDC locus **(Fig 5A and 5B).** CTCF and Rad21 showed high frequency of the binding in the gene-rich TAD1, TAD4, TAD6 and TAD7, while lower frequency of binding was seen in the TAD3 and TAD2 and lack of binding was detected in TAD5 **(Fig 5A and 5B).** We found CTCF binding within 100kb of all the TAD border midpoints, except the border between TADs 5 and 6, where it was found within 160kb **(Fig 5A)**, consistent with recently established role for CTCF in the TAD organization [9, 15, 21]. Similarly to CTCF and Rad21, Brg1 binding was abundant in all gene-rich, but not gene-poor TADs **(Fig 5A and 5B)**.

A substantial fraction of the 5C interactions showing CTCF, Rad21 and Brg1 binding (between 38% and 50% of all interactions for the individual proteins) **(Fig 5C)**, suggested that they might be involved in the control of the higher-order chromatin folding at the EDC locus in keratinocytes. Exact Fisher statistical test showed the enrichment for the regions bound by CTCF in all significant 5C looping interactions in comparison to all background 5C interactions **(Fig 5D).** This was consistent with a well-established role of CTCF in the control of higher-order chromatin folding in different cell types [4, 53, 54].

We further analyzed the pair-wise combinations of the chromatin architectural protein binding in the regions anchoring the 5C interactions at the EDC locus in keratinocytes. Consistently with the previously published data, our analysis revealed most frequent presence of the cohesin subunit Rad21 in the regions anchoring the 5C interactions that were also anchored to the CTCF binding regions (76.6%) (**Fig 5E)**. Brg1 was also frequently seen in the regions involved in the 5C interactions anchoring CTCF-bound regions (63.4%) **(Fig 5E).** CTCF, and Brg1 were present in the regions anchoring 59.4%, and 56.5% of the 5C interactions anchored to the Rad21 binding regions respectively **(Fig 5E).** These data demonstrates that CTCF, Rad21 and Brg1 frequently present in the regions anchoring the same 5C interactions, suggesting that they might functionally cooperate in the control of establishment of the spatial interacting network within the EDC locus and its genomic neighbourhood in keratinocytes.

Next we check if CTCF, Rad21, and Brg1 are involved in spatial contact between gene promoters and enhancers. We found that CTCF, Rad21 and Brg1 were even more frequently bound to the bases of the 5C loops involving gene enhancers than in the bases of all significant 5C loops **(Fig 5C and 5F)**. Exact Fisher statistical test demonstrated highly significant enrichment of this protein binding in the regions anchored to the enhancer spatial interactome (**Fig 5G)**, supporting their involvement in establishing promoter-enhancer contacts in keratinocytes. This is consistent with the role of Rad21 together with or independently from CTCF that has been well documented in several cell types [4, 53]. Moreover, Brg1 binding has also been reported to be frequently associated with active enhancers [55, 56], and promoter-enhancer spatial interactions [57]. Thus, our data suggest the important role for CTCF, Rad21 and Brg1 in organization of the 5C interactome within and between gene-rich TADs in the EDC locus in keratinocytes and in establishing promoter-enhancer spatial network in this locus.

## Discussion

Mouse genome contains 11 large multi-TAD gene loci, occupying >1.6 Mb each on the corresponding chromosomes, show a clustering of functionally related genes whose transcription is regulated in a lineage-specific manner [9, 27, 28]. In this manuscript, we demonstrate that in skin epithelial cells, EDC is organized into four TADs with the distinct chromatin interaction patterns within and between these and neighbouring TADs involving gene promoters and enhancers. We also show the promoter-enhancer anchoring regions in the gene-rich transcriptionally active TADs are enriched for the binding of chromatin architectural proteins CTCF, Rad21 and chromatin remodeler Brg1. In contrast to gene-rich TADs, gene-poor TADs show preferential spatial contacts with each other, do not contain active enhancers and show decreased binding of CTCF, Rad21 and Brg1 in keratinocytes.

The validation of the 5C data by 3D-FISH analyses performed according to the recommendations published previously [32] confirm that in epidermal keratinocytes, the central gene-rich EDC region, harbouring the majority of the genes activated during terminal keratinocyte differentiation, has two adjacent gene-rich TAD3 and TAD4, which are flanked by two gene-poor TAD2 and TAD5 further surrounded by the gene-rich TAD1, TAD6 and TAD7 **(Fig 2D).** Our 5C data at the EDC locus in keratinocytes are concordant with the data on the TAD organization identified by Hi-C approach in mouse embryonic stem cells and our 5C data in thymocytes used as a control in which EDC locus is largely inactive **(Figs 1B and 2D)** [9]. Some differences in the positions of the TAD borders between these datasets might reflect the differences in the resolution depth depicted by the 5C and Hi-C technologies, or real differences in the TAD borders between pluripotent (ground state of TAD organization in embryonic stem cells) versus differentiated cells. It remains to be determined whether these differences might also be linked to the distinct chromatin compartmentalization patterns in keratinocytes and thymocytes associated with striking differences in the EDC gene transcription between two cell lineages.

Combination of the 5C, 3D-FISH and ChIP-seq approaches reveal several differences between gene-rich and gene-poor TADs that constitute EDC locus and its neighbouring regions in epidermal keratinocytes. Gene-rich and gene-poor TADs within the locus show distinct inter-TAD spatial chromatin contact patterns. Gene-poor TADs (TAD2 and TAD5) and gene-rich TADs (TAD3 and TAD4) are compartmentalized in the nucleus as distinct topological domains, the transcriptionally inactive chromatin domains (compartment B) and active transcription (compartment A) [12, 45]. However, TAD2 and TAD5 show heterogeneity in their chromatin interaction patterns–TAD5 show preferential interactions with TAD2, while TAD2 also interacts with neighbouring gene-rich TAD3 and TAD4. In contrast to gene-rich TADs, gene-poor TADs do not contain active enhancers and show markedly decreased binding of CTCF, Rad21 and Brg1 proteins. Interestingly, the network of spatial interactions involving gene promoters and enhancers at the EDC locus in keratinocytes are not restricted to intra-TAD interactions, but the interactions extend to different gene-rich transcriptionally active TADs.

Our 5C data demonstrate that that majority (73.6%) of the “true” 5C contacts in 5.3 Mb chromatin domain in keratinocytes analysed in this study are mapped at sites near gene promoters and their interactions connect to non-promoter chromatin domains (47.3%) or to other promoters (26.3%). The promoter-promoter interactions are recently demonstrated using high-resolution capture Hi-C [49], and they are frequently identified by the ChIA-PET approach using anti-RNA polymerase II antibody [58]. The role of the promoter-promoter contacts in gene expression control is not well understood, however, promoters can share common transcription factories (foci enriched in RNA polymerase II) [59, 60], while some promoters can function as enhancers for their interacting promoter partners [58, 61].

Correlation of the 5C data with ChIPseq analyses for enhancer-specific histone modifications in KCs (high level of H3K4me1 and H3K27ac) reveal 16 putative active gene enhancers at the EDC locus in keratinocytes. Two of these enhancers (E9 and E11) **(Fig 3C)** were previously identified based on the non-coding region homology between several mammalian species and were shown to possess the enhancer activity in enhancer-reporter assay in cultured mouse keratinocytes [33]. About 52% of the significant 5C contacts involving gene enhancers show their interaction with the gene promoters, thus supporting a view on functional importance of such contacts identified in this study. However, further analyses are required to demonstrate functional relevance of these spatial contacts to the control of gene transcription in the epidermal progenitor cells and differentiating keratinocytes.

Intriguingly, in addition to the intra-TAD contacts, we demonstrate the extensive enhancer-promoter interactions across the TADs borders. Although less frequent, these contacts were longer-ranged (from 500 kb to 5.1 Mb) compared to the intra-TAD contacts (up to 600Kb). These data are consistent with recent reports demonstrating the presence of promoter-enhancer contacts across TAD boundaries in different cell types [16, 17, 19]. In cultured mouse keratinocytes, the recent 3C data identified interactions between the AP-1 dependent enhancer located in the TAD3 with several promoters within TAD3 and TAD4, as well as with *S100a6* promoter in TAD1 [37]. However, it is still unclear whether adjacent closely associated TADs can share regulatory elements by forming meta-TADs at large loci harbouring multiple co-regulated genes, similarly to the meta-TAD domains described in differentiating neuronal progenitor cells [18].

Interestingly, the enhancers found in TAD1 form two closely located clusters (E2/E3 and E4-E7), embedded into the genes of *S100* family. These enhancer clusters showed extensive long-range intra-TAD chromatin contacts with multiple genes in the central part of the EDC (TAD3 and TAD4) activated during terminal keratinocyte differentiation, suggesting that they might serve as the locus-control regions or super-enhancers for the EDC genes. In addition, we identified the gene enhancer (E9) spatially interacting with *Flg* gene promoter **(Fig 4D).** These enhancers have been previously identified among the highly-conserved non-coding regions in several mammalian genomes and showed the activity in the reporter assay in cultured keratinocytes [33]. It will be important to determine if this conserved enhancer controls *Flg* gene expression in normal and diseased epidermis, as the defects in *Flg* gene and changes in its expression are associated with ichthyosis vulgaris, the most common disorder of epidermal differentiation, and also serve as strong risk factors for atopic eczema [62].

The binding studies for chromatin architectural proteins CTCF, Rad21, and ATP-dependent chromatin remodeler Brg1 revealed the enrichment in the CTCF binding in the regions anchored to all significant 5C contacts. In particular, binding of the CTCF, cohesin complex subunit Rad21 and ATP-remodeller Brg1 was enriched in the regions anchoring the 5C interactions involving gene enhancers within gene-rich TADs. These findings are consistent with the well-established roles of CTCF and cohesin complex in the control of spatial genome topology [11, 13, 20, 22]. Recent Hi-C data from Khavari’s lab on human keratinocytes also revealed a role for Rad21 in the control of enhancer-promoter contacts in both progenitors and differentiated cells (J Invest Dermatol, 2017, 137, 5S, S80, abstract). In addition, Brg1 is frequently found at the gene enhancers [55, 56] and it was reported to be involved in the enhancer-promoter looping interactions [57, 63]. However, since CTCF and cohesin are ubiquitously expressed across the broad range of cell types, suggesting that additional proteins with more restricted expression patterns might be involved in shaping lineage-specific spatial genome organization.

Taken together, our findings provide new insights into the spatial chromatin organization at the large multi-TAD EDC locus with extensive spatial contacts involving gene promoters and enhancers within and between different gene-rich TADs. Such interactions might contribute to the coordinated gene regulation in the EDC locus during terminal keratinocyte differentiation in the epidermis. These data serve as an important platform for future studies to reveal the intricate interplay between the chromatin architectural protein, chromatin remodelers, transcription factors and gene regulatory elements in the control of spatial genome organization and gene expression programmes in basal and differentiating epidermal keratinocytes during normal skin development and homeostasis, as well as during skin responses to environmental stressors and in disorders of epidermal differentiation, such as atopic dermatitis, psoriasis and cancers.

## Materials and methods

### Experimental animals and tissue collection

All animal studies were performed under protocol approved by the University of California Berkley Institutional Animal Care and Use Committee and the UK Home Office Project Licence. C57Bl/6 mice were purchased from Charles River. The skin tissue samples were collected from P1.5-P3.5 C57Bl/6 animals as previously described [64, 65]. Keratinocytes were isolated for micro-array, 5C, 3D FISH and ChIP-seq analysis from the skin of the new born C57BL/6 animals. Primary thymocytes were isolated from the C57Bl/6 animals. For the FISH analysis of 3D preserved nuclei, skin samples were processed as previously described [66, 67]]

### Isolation of primary epidermal keratinocytes and thymocytes

Primary epidermal keratinocytes was isolated from the skin of the new-born C57Bl/6 mice as previously described [68, 69]. Briefly, the skin was removed from the neonatal mice and incubated with 0.25% trypsin in Hanks Balanced Salt solution overnight at 4 C, followed by separation of dermis from epidermis. Epidermis was placed into pre-chilled low calcium primary keratinocyte culture (EMEM, 4% chelated FBS, 0.05mM CaCl_2_, 0.4ug/ml hydrocortison, 5ug/ml insulin, 10mg/ml EGF, 10^−10^ M cholera toxin, 2x10^-9^ T3, 2mM L-glutamin, 100U/ml penicillin, 100ug/ml streptomycin) and triturated to obtain the single cell suspension. The cells were filtered through a 70 μm silicon strainer and were either seeded at high density at the low calcium primary keratinocyte medium onto collagen solution (0.97X Hanks Balanced Salt Solution (HBSS), 9.70μg/mL Bovine Serum Albumin (BSA), 19.40 mM 4-(2-hydroxyethyl)-1-piperazineethanesulfonic acid (HEPES), 0.97 X Vitrogen-100 Collagen) coated culture dishes for 15 hours at 32°C in the atmosphere of 8% carbon dioxide and 90% humidity, or were used for FACS to isolated viable basal keratinocyte population.

Primary thymocytes were isolated from C57Bl/6 mouse thymi as described in [70]. The thymi were transferred into pre-chilled T cell medium (RPMI medium 1640 (ATCC modification), 10% foetal bovine serum, 0.1x 2-mercaptoethanol) and crushed to release total thymus T cell population. The cells were filtered through a 70 μm cell strainer, pelleted by centrifugation and re-suspended in Red Blood Cell lysis buffer (Sigma) for 3 min. Cell were then washed with the pre-chilled T-cell medium, re-suspended in the medium, filtered through a 70 μm cell strainer and counted using haemocytometer.

### RNA isolation, micro-array and qRT-PCR analysis

RNA was isolated from the primary keratinocytes plated on the collagen solution coated dishes for 15 hours at 32°C and 8% CO_2_ or primary thymocytes using TRI Reagent solution and TURBO-DNA-free kit (Invitrogen). Total RNA was amplified with Arcturus Ribo-Amp PLUS system (Applied Biosystems) as previously described [36]. RNA was converted into labelled cDNA and micro-array analysis was performed by MoGene (St Louis, MO, USA) using 41K Whole Mouse Genome 60-mer oligo micro-arrays (Aglinent Technologies). Micro-array datasets were analysed using the distribution of background intensity and signal intensity values (Agilent Feature Extraction software version 7.5).

### 3C template generation and characterization

Two 3C templates were constructed for freshly plated epidermal keratinocytes and primary thymocytes according to [71] with modifications. Briefly, epidermal keratinocytes isolated from mouse epidermis were seeded in the low calcium primary keratinocyte medium at high density on the collagen coated plate for 15 hours at 32°C, 8% CO_2_ and 90% humidity. The primary thymocytes were isolated as described above. Cells were washed twice with the growth medium and fixed with 1% formaldehyde (Electron Microscopy Systems) in the growth medium for 10 minutes at room temperature with gentle mixing every 2 minutes. The glycine was added to a final concentration of 125 mM. Quenching was initiated at room temperature and the cells were placed on ice for 5 min. The medium was removed and cells were washed ones with ice cold PBS and then fresh ice cold PBS was added. Cross-linked cells were collected, counted, pelleted by centrifugation in aliquots and quick-frozen. Cells were stored at -80°C.

Per a 5C library, the frozen pallet of 6x10^7^ cells 1.2 ml of lysis buffer (10 mM Tris-HCl, pH 8.0, 10 mM sodium chloride, 0.2% (vol/vol) Igepal C-630 (Sigma)) supplemented with 120 ul of protease inhibitor cocktail (Sigma) was added and cells were incubated on ice for 30 minutes. Cells were lysed using a 5 ml dounce homogenizer, washed twice with ice cold 1x NEBuffer2 buffer (10 mM Tris-HCl, 50 mM NaCl, 10 mM MgCl2, 1 mM DTT), and re-suspended in 630 ul of 1x NEBuffer2. Nuclear suspension was divided into 50 ul aliquots. To the nuclear suspension 312 ul of 1xNEBuffer2 was added. SDS was then added to a final concentration of 0.1% and lysates were incubated at 65°C for 10 min. Triton X-100 was then added to a final concentration of 1% to quench SDS. To each aliquot of solubilized chromatin 800 U of HindIII enzyme (New England Biolabs) was added and the digestion was performed overnight at 37°C with shaking. HindIII was inactivated by incubating lysates at 65°C for 30 min after addition of SDS to a final concentration of 1.56%. Ligation was performed under diluted conditions that promote intra-molecular ligation at 16°C for 4 hr in ligation buffer (1% Triton X-100, 0.1 mg/ml BSA, 1 mM ATP, 50 mM Tris-HCl (pH 7.5]) 10 mM MgCl2, 10 mM DTT) with 10 ul of T4 DNA ligase (Invitrogen). To reverse crosslinks, samples were then treated with 63.5 mg/ml Proteinase K (Invitrogen) at 65°C. Four hours later, Proteinase K was added again to 127 mg/ml and then incubated overnight at 65°C. DNA was purified by subjecting samples to a series of phenol and phenol-chloroform extractions before precipitation with ethanol. Pellets were re-suspended in 1–2 ml TE Buffer, pH8.0 and precipitation with ethanol. Pellets were re-suspended in 500 ul of TE buffer and treated with DNase-free RNase at final concentration of 100 ng/ul for 1 hour at 37°C. 3C templates were further purified using Amicon Ultra Centrifugal 30K Filter for DNA Purification and Concentration (Millipore). Using the Millipore columns, samples were washed twice with 1X TE buffer. Following sample recovery from Millipore columns, initial sample volume was then restored with 1X TE buffer, pH 8.0.

The concentration of the 3C template was assessed by gel electrophoresis with high molecular weight DNA ladder as a standard (Invitrogen) using TotalLab Quant gel densitometry software. Controls for DNA integrity (undigested chromatin control) and restriction digestion (no ligase control) were also checked and passed the quality control. The quality of the 3C templates were further assessed by running the PCR with series of 2-fold dilutions of the templates with forward (ATGGAGACCTGCCGCCGGCTCATCACAC) and reverse (CGTGCTGTGACTTCGCACTTTTCTGATC) primers amplifying the product of head to head ligation of two HindIII sites located 1164bp apart as described in [71] using Quant gel densitometry software (Total lab).

### 5C library construction and sequencing

Two independent 5C libraries were constructed for each cell type as described in [71] with modifications. 5C probes were designed at HindIII restriction sites using the my5Csuite primer design tools [44]. An alternating scheme was pursued in which reverse and forward probes were designed against every other fragment. Probes were excluded if unique mapping could not be achieved for fragments spanning highly repetitive sequences. Probe setting were as follows: U-BLAST, 3; S-BLAST, 50; MER, 800; MIN, FRAGSIZE, 100; MAX FRAGSIZE, 50000; OPT_TM, 65: and OPT_PSIZE, 40. The universal T7 sequence was tethered to all forward primers (TAATACGACTCACTATAGCC) and the reverse complement to the universal T3 sequence was tethered to all reverse probes (TATTAACCCTCACTAAAGGGA). In total, 381 forward probes and 382 reverse probes were designed, spanning 5.3 Mb EDC containing locus (**Fig 1B, S2 Table**).

To construct 5C libraries, first probes were annealed to the 3C templates at 48°C for 16 hours. Each multiplex annealing reaction contained 1xNEBuffer4 (New England Biolabs), 560 ng of 3C template and 0.4 fmole of each 5C probe. The annealed probes were nick ligated with 10 U of Taq ligase in 1x Taq ligase buffer (New England Biolabs) at 48°C for 1 hour. The resulting 5C library was amplified by PCR with 25 cycles using universal T7 (TAATACGACTCACTATAGCC) and T3 (TATTAACCCTCACTAAAGGGA) primers. 15 ligation reactions amplified in 6 PCR reactions each were performed to generate each 5C library. The PCR reactions for each 5C library were pooled before further processing. 5C library amplification reactions produced the products of expected size (101 bp), while the negative control PCR reactions (included no 5C template control, no ligation control or no 5C probe control) did not yield any PCR product.

5C libraries were size fractioned (101 bp) and purified from the agarose gel using QIAquick gel purification kit (QIagene). 3’ A-tails were added using dATP and Taq polymerase, followed by subsequent ligation to bar-coded custom designed adaptor oligonucleotide [72] for Illumina pair-end sequencing. Adaptor-modified 5C libraries were purified after separation in the agarose gel using QIAquick gel purification kit (Qiagene). The purified libraries were amplified by 18 cycles of PCR with PE1.0 and PE2.0 primers (Illumina). The amplified libraries (233 bp) were purified from the agarose gel, quantified using Nanodrop 1000 spectrophotometer (Thermo Fisher) and send for the sequencing on the HiSeq 2000 system at the EMBL Genome Core Facility (Heidelberg, Germany).

### 5C data processing and normalization

The 5C library sequencing data sets were de-multiplexed using Novobarcode (Novocraft). The reads were aligned to the pseudo-genome consisting of all 5C probes **(S2 Table)** using Bowtie [73]. To account for poor quality reads, sequences were required to have only one unique alignment. After mapping, interactions were counted when both paired end reads could be uniquely mapped to the 5C probe pseudo-genome. Only interactions between forward-reverse probe pairs were considered as true counts.

Next, we performed the data correction to remove the technical biases associated with the 5C technology as described in [17, 43] with some modifications. First, we removed the probes that performed significantly differently in comparison to the overall probe sets. A global average relationship between interaction frequency and genomic distance was calculated using Loess smoothing for each replicate dataset. Contact profile for each probe across the interrogated region was compared to this average. We removed the probes with the individual Loess of more or less than 0.85 of the scaled Z score distance from the global Loess. We removed 38 probes for the replicate 1 and 37 probes for the replicate 2 for the downstream analysis **(S15 Table, S2B, S3B, S4B and S5B Figs).**

After this step, we removed the signal interaction with very high contact frequency in comparison to their neighbors. We removed such interactions if they have a Z score of 25 or more **(S16 Table, S2C, S3C, S4C and S5C Figs)**. Z score was calculated as described in [17]. Finally, we normalized the profile of each probe so they could be quantitatively compared to each other as described in [17], but we calculated a global average relations between interaction frequency and genomic distance with Loess smoothing for each replicate separately **(S2D, S3D, S4D and S5D Figs)**.

### TAD boundary position identification using insulation index analysis

TAD boundary positions were identified by calculating an insulation score along the locus as described in [17, 46]. The normalized 5C data were binned at 150 kb with 15 kb step size. Next, we calculated the combined number of interactions across each bin by summing all interactions up to 500 kb upstream of the bin and up to 500 kb downstream of the bin. The sum for each bin was divided by the average sum for all bins to yield insulation score. The insulation score was plotted along the whole locus to obtain an insulation profiles **(Fig 2D, S6A Fig)**. Local minima in these profiles indicate the position of the TAD boundaries. The local minima in the insulation profile were detected by identifying the bins with the lowest insulation score in a local 435 kb window. The mid-points of these bins were set as the TAD boundaries. The average position of the midpoint between the replicate was used as the TAD boundaries in the manuscript **(S3 Table)**.

### Identification of the significant 5C interactions

To detect the “true” statistically significant chromatin looping interactions between the individual restriction fragments, we applied a “5 C peak calling approach as described before [17, 43]. We called the significant 5C peaks for the 5C libraries separately. Peaks were defined as normalized ligation frequencies (signals) that are significantly higher than expected for the genomic distances separating the interacting fragments. Expected values were calculated as the average interaction frequency for each genomic distance by using Loess smoothing (alpha value 0.01). This provides a weighted average and a weighted standard deviation for each genomic distance. We assumed that the large majority of interactions were not significant looping contacts, and we interpreted these weighted averages as the expected interaction frequencies for given genomic distances. We then transformed observed 5C interaction frequencies into a Z score by calculating the (observed value-expected value)/standard deviation. The calculated Z score distribution was fit to a Weibull distribution. p values were calculated for each Z score and transformed into q values for false discovery rate analysis. We used q-value threshold of 0.05 for the 5C peak calling. Only 5C peaks reproducible in both replicates in KCs or TCs were used for subsequent analysis **(S6 and S7 Tables)**.

### 3D FISH, image acquisition and data analysis

3D FISH analysis of the spatially preserved nuclei in the mouse skin tissue and freshly isolated primary keratinocytes [74] and thymocytes was performed as previously described with modifications [36, 67, 74, 75]. Primary keratinocytes were seeded overnight on the collagen coated cover slips. The adherent cells were fixed with formaldehyde and prepared for 3D FISH as described in [75]. Primary thymocytes were seeded on the slides coated with 1 mg/ml of Poly-L-lysine hydrobromide per [75]. 20 μm sections of the frozen skin sample with structurally preserved nuclei were used for the 3D analysis.

BAC based probes were prepared for the selected regions **(S4 Table)** by nick-translation using in house synthesized Bio-dUTP, FITC-dUTP, Cy3-dUTP or Dig-dUTP as described in [76]. After hybridization the samples were stained with Cy5-streptavidin or anti-Dig-Cy3 antibodies (**S17 Table)** when needed. DNA was stained with DAPI (Sigma). 3D images were collected using a Zeiss LSM510 confocal microscope. Nuclei were scanned with a z-axial distance of 200 nm, yielding separate stacks of 8-bit grey scale images, with pixel size 100–200 nm, for each fluorescent channel. For each optical section, images were collected sequentially for all fluorophores and the axial chromatic shift corrected for in each channel as described in [77]. Images were processed and analyzed using ImageJ (NIH). Inter-locus distances were calculated after correction for chromatic aberration, as previously described [36]. The differences between the inter-locus distances in different samples were analyzed using Mann-Whitney U-test.

### ChIP-seq analysis

For ChIP analysis new born C57Bl6 total keratinocyte single cell suspension was prepared as described above. To ensure analysis of viable cells with intact chromatin, keratinocytes were stained with UV Live/Dead Fixable Dye (Life Technologies) for 30 min on ice prior fixation with 1% PFA for 10 min at RT. Fixed cells were labeled with CD49f-PE and Sca-1-FITC antibodies **(S17 Table)** for 1 hour on ice. CD49f+/Sca-1+ basal keratinocytes were gated after exclusion of dead (UV Live/Dead Fixable Dye-positive, Life Technologies) cells and sorted on a MoFlo XDP cell sorter (Beckman Coulter), as described in [78]. Sorted cells were pelleted at 2.000 g and stored at -80°C.

ChIP was performed using FACS sorted epidermal keratinocytes isolated from newborn mouse skin anti-H3K27ac, anti-Rad21 and anti-CTCF antibodies **(S17 Table)** using ChIP-IT High Sensitivity kit (Active Motif) as described in [36, 79].

ChIP with anti-H3K4me1 antibodies **(S17 Table)** was performed using Micrococcal nuclease (MNase) digestion epidermal keratinocyte chromatin as per [80]. 1x10^6^ cells were used per MNase digestion and 1μg of the antibodies per IP comprising pre-cleared chromatin corresponding to 5x10^5^ cells.

Indexed ChIP-Seq libraries from immune-precipitated and control input chromatin were generated using NEBNext ChIP-Seq Library Prep Master Mix Set (New England BioLabs) for Illumina and NEBNext Multiplex Oligos for Illumina (New England BioLabs).

The libraries were sequenced on the HiSeq 2500 system (Illumina), producing 30–70 million reads per library. Sequencing reads were aligned to the mm9 mouse genome assembly [73]. Specific areas of protein binding or histone modification presence were identified with MACS using default parameters [81]. The normalized ChIP-seq signals together with the previously published ChIP-seq signals for Brg1 were visualized using UCSC genome browser (http://genome.ucsc.edu) [82, 83].

### Pursing the putative enhancers in keratinocytes

High confidence H3K4Me1 ChIP-seq peaks were merged if they were located within 5 kb end-to-end distances from each other and the same operation was applied to the H3K27ac ChIP-seq peaks. Enhancers were defined as merged H3K4me1 and H3K27ac peaks located within 2 kb end-to-end distance. We did not exclude putative enhancers located near gene promoters, as recent studies indicate that gene promoters could poses gene enhancer activity and enhancers could be located close to gene promoters [58, 61]. The positions of the gene enhancers were visualized using UCSC genome browser (http://genome.ucsc.edu) [82, 83].

### Analysis of 5C interactomes for enrichment of the binding of non-histone chromatin proteins in the anchoring regions

An enrichment of selected non-histone protein binding in the regions anchoring 5C interactions at EDC locus or interactions involving gene enhancers at the locus were calculated for the extended 5C fragments to include nearest adjacent fragments interrogated by the 5C probes on the opposite strand as described in [11].

## Supporting information

S1 Fig **(a)** Relative mRNA expression levels in freshly plated murine keratinocytes and thymocytes aligned to the schematic map of the 5,3 Mb locus analyzed using 5C technologyin this study. **(b)** Heatmaps representing raw 5C data for both TC replicates. Reverse probes are plotted as columns and the forward probes as rows. Pearson’s correlation coefficient is also shown.
(TIF)

S2 Fig 5C data correction and normalization for the keratinocyte library replicate 1. In all heatmaps the reverse probes shown in columns and forward probes shown in rows. **(a)** Raw data **(b)** Data after 5C probe cis-purge. Grey stripes represent probes that were removed **(c)** Data after singleton interaction removal. Grey stripes are the primers removed in the previous step, grey pixels are individual interactions removed in this step. **(d)** Final coverage corrected data. Grey lines and pixel represent all the 5C probes and the individual interactions removed at previous steps and at this step. **(e)** Binned raw and coverage corrected data (bin size 150kb, step size 15kb). Grey lines and pixels indicate the regions lacking data due to the poor probe coverage or removed signals after correction.
(TIF)

S3 Fig 5C data correction and normalization for the keratinocyte library replicate 2. In all heatmaps the reverse probes shown in columns and forward probes shown in rows. **(a)** Raw data **(b)** Data after 5C probe cis-purge. Grey stripes represent probes that were removed **(c)** Data after singleton interaction removal. Grey stripes are the primers removed in the previous step, grey pixels are individual interactions removed in this step. **(d)** Final coverage corrected data. Grey lines and pixel represent all the 5C probes and the individual interactions removed at previous steps and at this step. **(e)** Binned raw and coverage corrected data (bin size 150kb, step size 15kb). Grey lines and pixels indicate the regions lacking data due to the poor probe coverage or removed signals after correction.
(TIF)

S4 Fig 5C data correction and normalization for the thymocyte library replicate 1. In all heatmaps the reverse probes shown in columns and forward probes shown in rows. **(a)** Raw data **(b)** Data after 5C probe cis-purge. Grey stripes represent probes that were removed **(c)** Data after singleton interaction removal. Grey stripes are the primers removed in the previous step, grey pixels are individual interactions removed in this step. **(d)** Final coverage corrected data. Grey lines and pixel represent all the 5C probes and the individual interactions removed at previous steps and at this step. **(e)** Binned raw and coverage corrected data (bin size 150kb, step size 15kb). Grey lines and pixels indicate the regions lacking data due to the poor probe coverage or removed signals after correction.
(TIF)

S5 Fig 5C data correction and normalization for the thymocyte library replicate 2. In all heatmaps the reverse probes shown in columns and forward probes shown in rows. **(a)** Raw data **(b)** Data after 5C probe cis-purge. Grey stripes represent probes that were removed **(c)** Data after singleton interaction removal. Grey stripes are the primers removed in the previous step, grey pixels are individual interactions removed in this step. **(d)** Final coverage corrected data. Grey lines and pixel represent all the 5C probes and the individual interactions removed at previous steps and at this step. **(e)** Binned raw and coverage corrected data (bin size 150kb, step size 15kb). Grey lines and pixels indicate the regions lacking data due to the poor probe coverage or removed signals after correction.
(TIF)

S6 Fig **(a)** Heatmap representing the 5C data after the normalization and binning (bin size 150 kb, step size 15kb) in TCs. The position of TAD border midpoints (average for the midpoints calculated based on the insulation index analysis in two replicates independently) are identified by green lines under the heatmaps. Schematic map of the studied locus and insulation indexes profiles for two 5C library replicates are also shown. **(b)** Box plots showing median, 25% quartile, 75% quartile with whiskers indicating maximum and minimum for spatial distances between the centres of the regions covered by probes A and B, and probes B and C **(Fig 2D)** before (in μm) and after normalization to the average nuclear radius (in percentage of average nuclear radius) in freshly plated primary KCs and TCs (used to prepare 5C libraries). The distances between the centres of the regions covered by the probes A and B (located within TAD3) are significantly shorter than the distances between loci covered by the probes B and C (located within TAD4) in two cell types. The indicated p-values for pair-wise comparison are calculated using Mann-Whitney U-test, n = 60 alleles for each locus. Note, that the corresponding distances in TCs are significantly longer (p-value <0.0001) than in keratinocytes. **(c)** Vent diagram indicating the overlap of the significant 5C interactions (q<0.05) between the 5C library replicates in TCs. **(d)** Vent diagram showing KC specific “true” 5C interactions (blue), TC specific “true” 5C interactions (red) and “true” 5C interactions common in both cell types (green). **(e)** Number of the significant 5C interactions between and within the individual TADs. **(f)** Scaling plot showing log10 of the average normalized read counts versus log10 for genomic distances separating the contacting regions for the whole data set (black), intra-TAD contacts (red) and inter-TAD contacts (blue).
(TIF)

S1 Table Micro-array expression data in keratinocytes and thymocytes. (XLSX)

S2 Table 5C probe sets. (XLSX)

S3 Table TAD boundaries. (XLSX)

S4 Table BAC probes used for the 3D FISH analysis. (XLSX)

S5 Table 3D FISH inter-probe distances. (XLSX)

S6 Table All significant 5C looping interactions reproducible in both keratinocyte library replicates. (XLSX)

S7 Table All significant 5C looping interactions reproducible in both thymocyte library replicates. (XLSX)

S8 Table Keratinocyte-specific 5C interactions. (XLSX)

S9 Table Thymocyte-specific 5C interactions. (XLSX)

S10 Table 5C interactions common in keratinocytes and thymocytes. (XLSX)

S11 Table Gene enhancers identified by H3K4me1 and H3K27ac ChIP-seq. (XLSX)

S12 Table All 5C interactions involving gene enhancers. (XLSX)

S13 Table 5C interactions between gene promoters and enhancers. (XLSX)

S14 Table 5C interactions between gene enhancers and promoters in different TADs. (XLSX)

S15 Table 5C probes removed in probe filtering step. (XLSX)

S16 Table Individual interactions removed in singleton removal step. (XLSX)

S17 Table Antibodies. (XLSX)
