# Supplementary figures and images for: 5C analysis of the Epidermal Differentiation Complex locus reveals distinct chromatin interaction networks between gene-rich and gene-poor TADs in skin epithelial cells
Source: PLoS Genet. 2017 Sep 1;13(9):e1006966. doi: 10.1371/journal.pgen.1006966 (PMC5599062; doi:10.1371/journal.pgen.1006966)

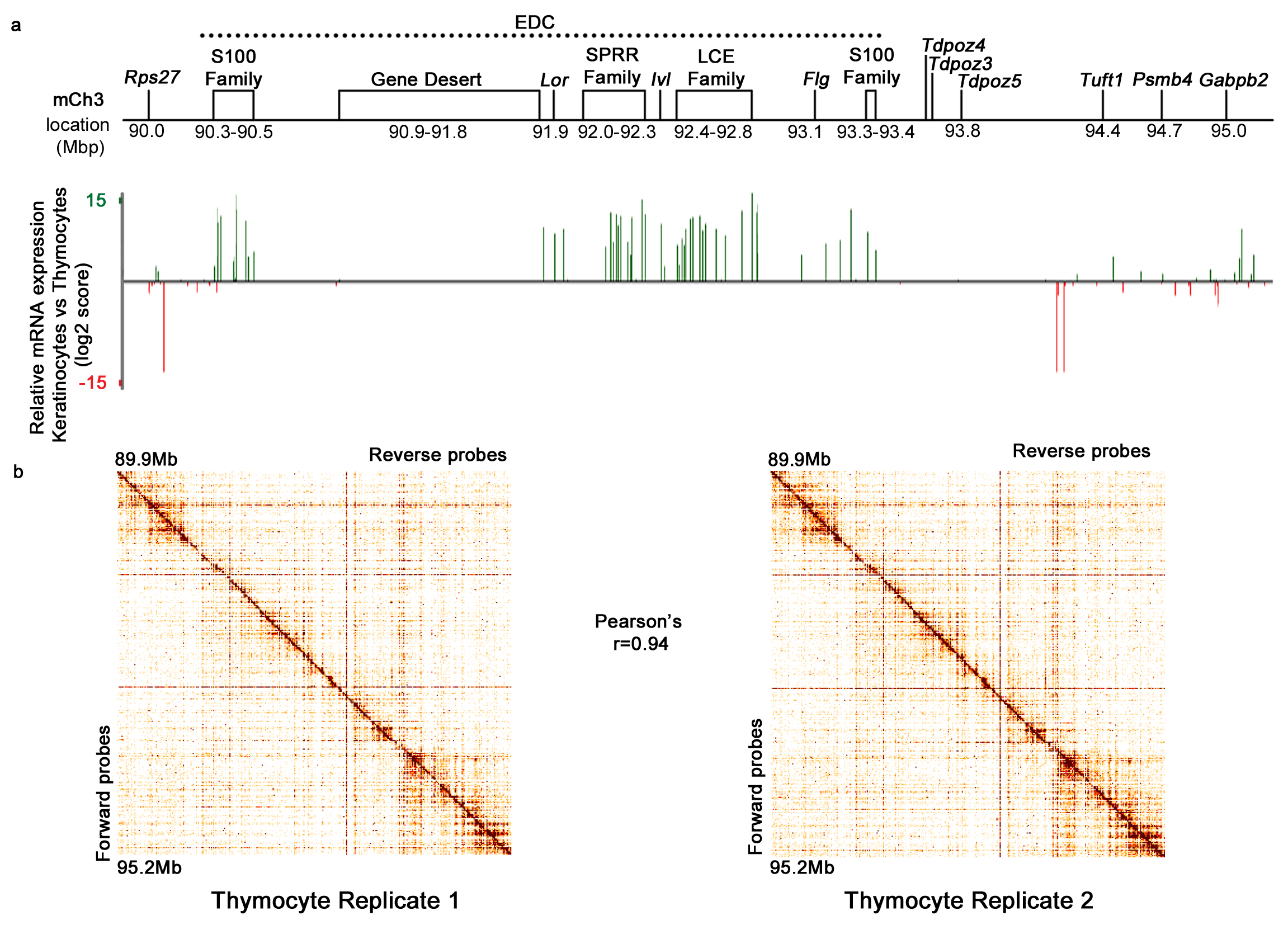

Supplement: S1 Fig — (a) Relative mRNA expression levels in freshly plated murine keratinocytes and thymocytes aligned to the schematic map of the 5,3 Mb locus analyzed using 5C technologyin this study. (b) Heatmaps representing raw 5C data for both TC replicates. Reverse probes are plotted as columns and the forward probes as rows. Pearson’s correlation coefficient is also shown. (TIF) [file pgen.1006966.s001.tif]

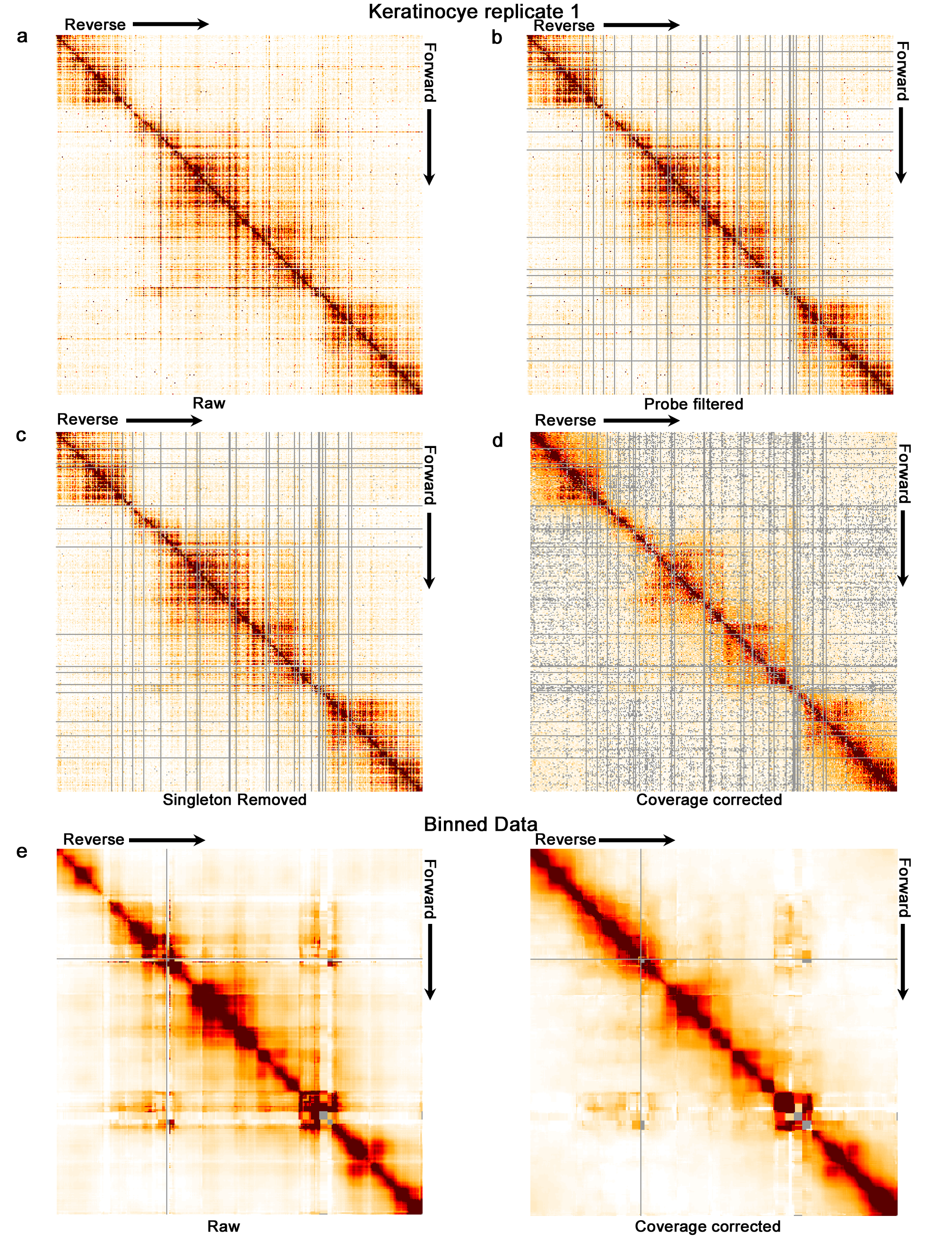

Supplement: S2 Fig — In all heatmaps the reverse probes shown in columns and forward probes shown in rows. (a) Raw data (b) Data after 5C probe cis-purge. Grey stripes represent probes that were removed (c) Data after singleton interaction removal. Grey stripes are the primers removed in the previous step, grey pixels are individual interactions removed in this step. (d) Final coverage corrected data. Grey lines and pixel represent all the 5C probes and the individual interactions removed at previous steps and at this step. (e) Binned raw and coverage corrected data (bin size 150kb, step size 15kb). Grey lines and pixels indicate the regions lacking data due to the poor probe coverage or removed signals after correction. (TIF) [file pgen.1006966.s002.tif]

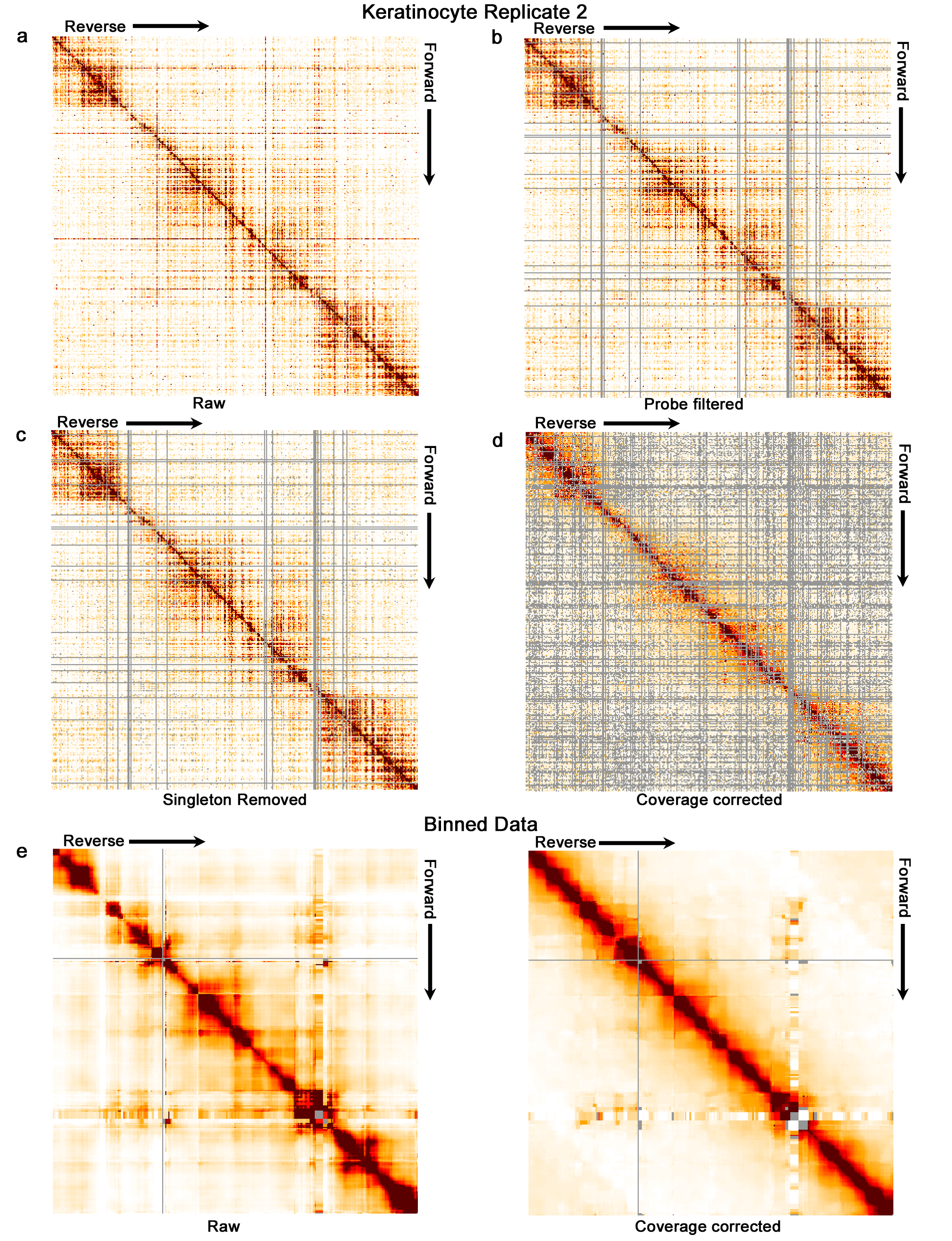

Supplement: S3 Fig — In all heatmaps the reverse probes shown in columns and forward probes shown in rows. (a) Raw data (b) Data after 5C probe cis-purge. Grey stripes represent probes that were removed (c) Data after singleton interaction removal. Grey stripes are the primers removed in the previous step, grey pixels are individual interactions removed in this step. (d) Final coverage corrected data. Grey lines and pixel represent all the 5C probes and the individual interactions removed at previous steps and at this step. (e) Binned raw and coverage corrected data (bin size 150kb, step size 15kb). Grey lines and pixels indicate the regions lacking data due to the poor probe coverage or removed signals after correction. (TIF) [file pgen.1006966.s003.tif]

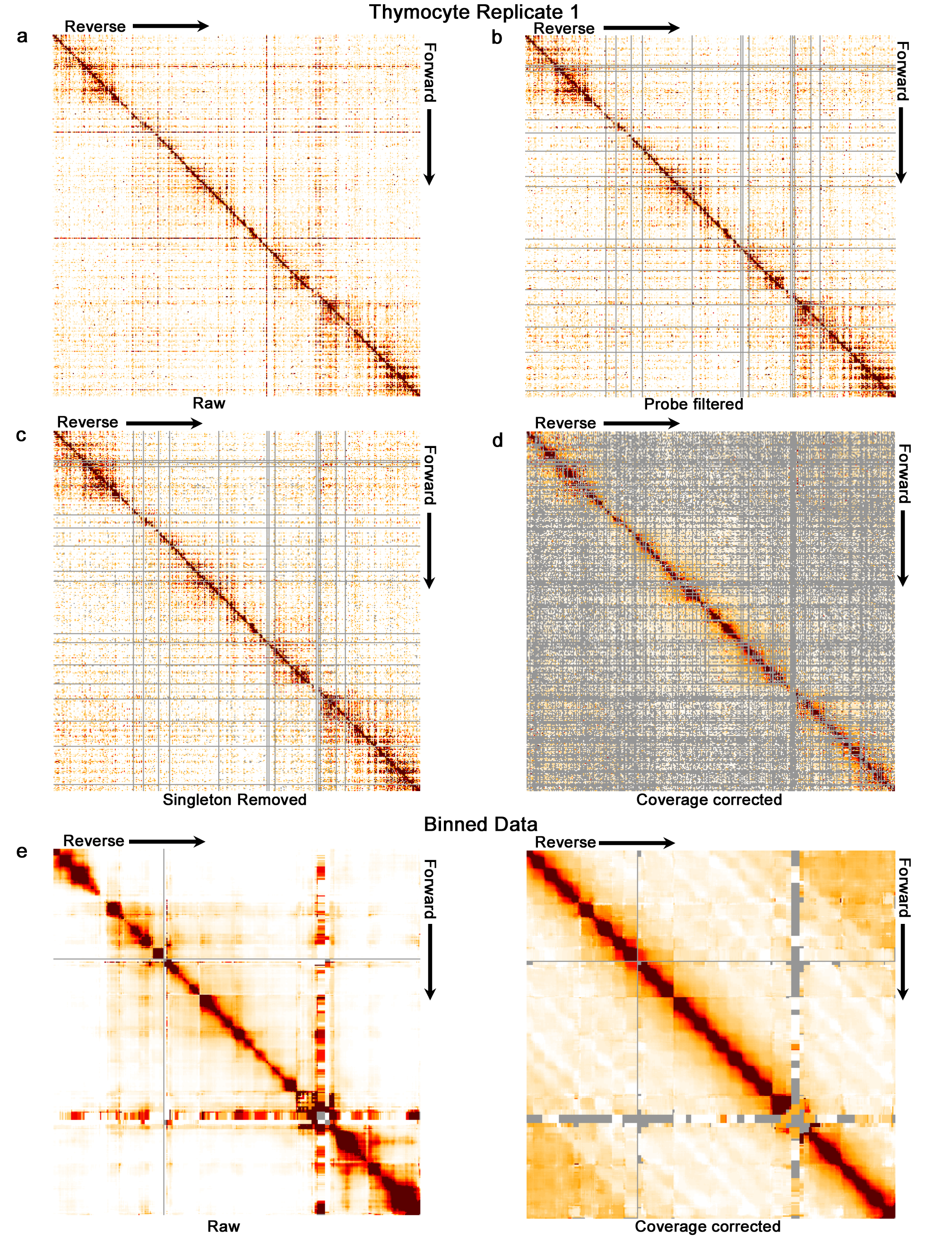

Supplement: S4 Fig — In all heatmaps the reverse probes shown in columns and forward probes shown in rows. (a) Raw data (b) Data after 5C probe cis-purge. Grey stripes represent probes that were removed (c) Data after singleton interaction removal. Grey stripes are the primers removed in the previous step, grey pixels are individual interactions removed in this step. (d) Final coverage corrected data. Grey lines and pixel represent all the 5C probes and the individual interactions removed at previous steps and at this step. (e) Binned raw and coverage corrected data (bin size 150kb, step size 15kb). Grey lines and pixels indicate the regions lacking data due to the poor probe coverage or removed signals after correction. (TIF) [file pgen.1006966.s004.tif]

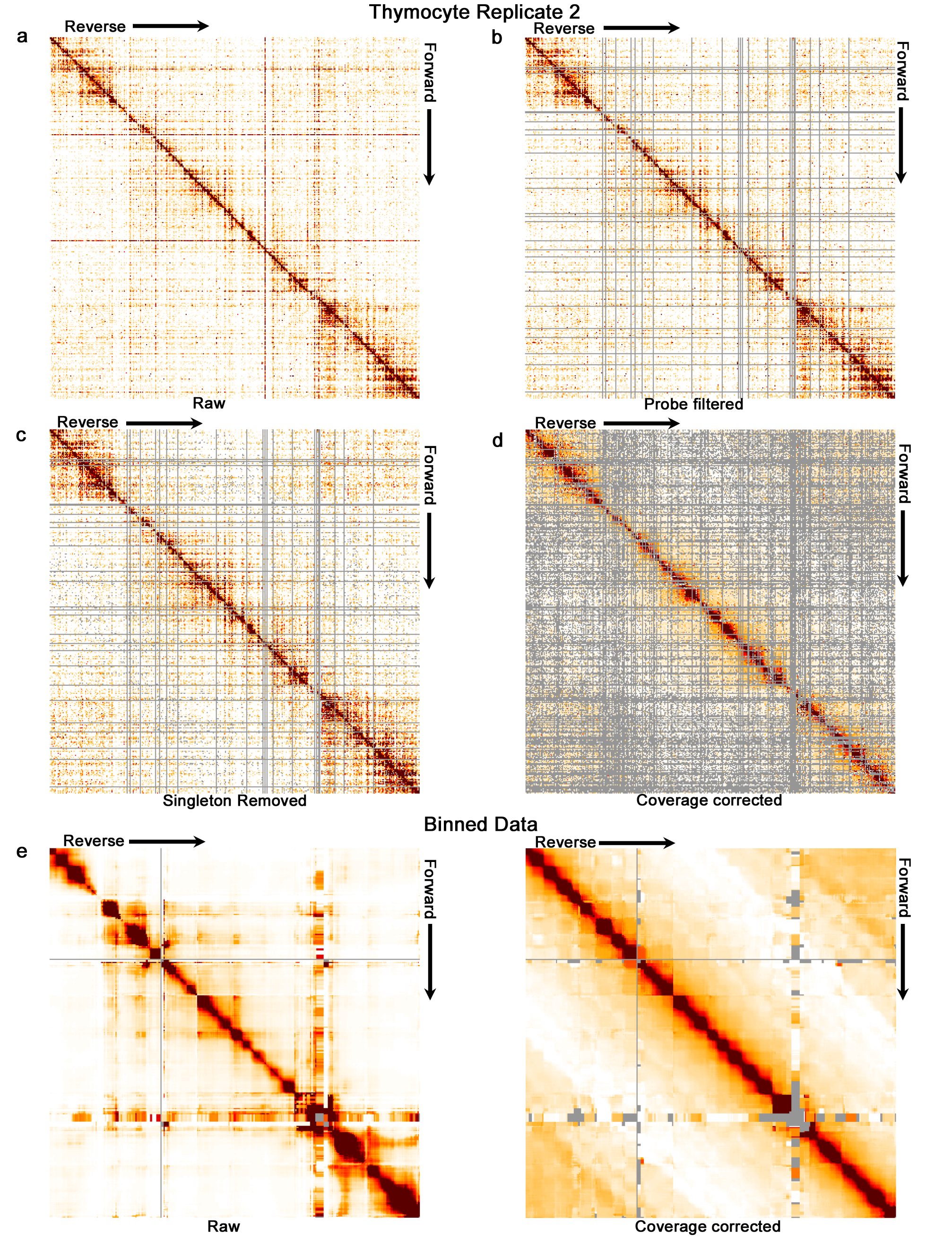

Supplement: S5 Fig — In all heatmaps the reverse probes shown in columns and forward probes shown in rows. (a) Raw data (b) Data after 5C probe cis-purge. Grey stripes represent probes that were removed (c) Data after singleton interaction removal. Grey stripes are the primers removed in the previous step, grey pixels are individual interactions removed in this step. (d) Final coverage corrected data. Grey lines and pixel represent all the 5C probes and the individual interactions removed at previous steps and at this step. (e) Binned raw and coverage corrected data (bin size 150kb, step size 15kb). Grey lines and pixels indicate the regions lacking data due to the poor probe coverage or removed signals after correction. (TIF) [file pgen.1006966.s005.tif]

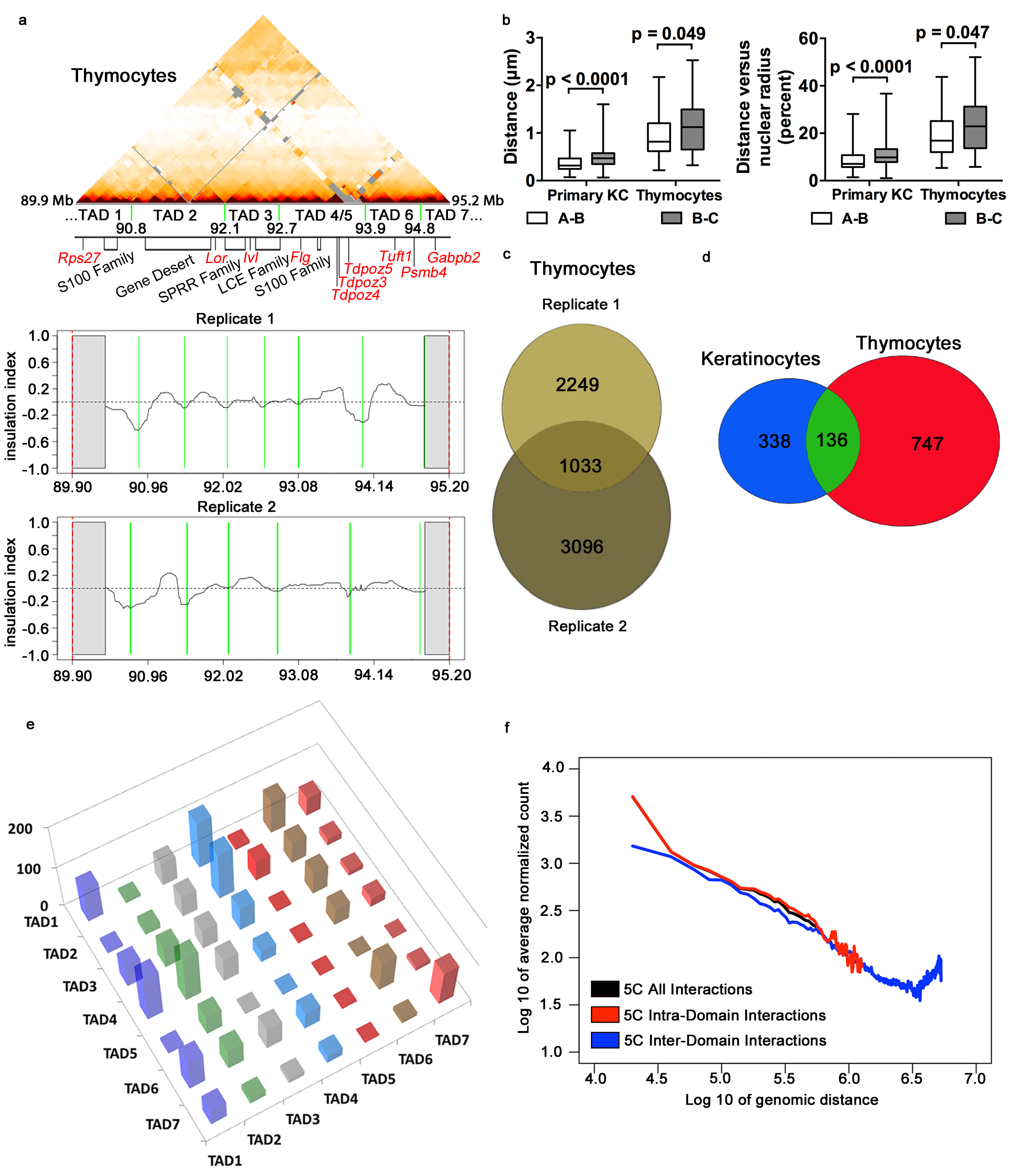

Supplement: S6 Fig — (a) Heatmap representing the 5C data after the normalization and binning (bin size 150 kb, step size 15kb) in TCs. The position of TAD border midpoints (average for the midpoints calculated based on the insulation index analysis in two replicates independently) are identified by green lines under the heatmaps. Schematic map of the studied locus and insulation indexes profiles for two 5C library replicates are also shown. (b) Box plots showing median, 25% quartile, 75% quartile with whiskers indicating maximum and minimum for spatial distances between the centres of the regions covered by probes A and B, and probes B and C (Fig 2D) before (in μm) and after normalization to the average nuclear radius (in percentage of average nuclear radius) in freshly plated primary KCs and TCs (used to prepare 5C libraries). The distances between the centres of the regions covered by the probes A and B (located within TAD3) are significantly shorter than the distances between loci covered by the probes B and C (located within TAD4) in two cell types. The indicated p-values for pair-wise comparison are calculated using Mann-Whitney U-test, n = 60 alleles for each locus. Note, that the corresponding distances in TCs are significantly longer (p-value <0.0001) than in keratinocytes. (c) Vent diagram indicating the overlap of the significant 5C interactions (q<0.05) between the 5C library replicates in TCs. (d) Vent diagram showing KC specific “true” 5C interactions (blue), TC specific “true” 5C interactions (red) and “true” 5C interactions common in both cell types (green). (e) Number of the significant 5C interactions between and within the individual TADs. (f) Scaling plot showing log10 of the average normalized read counts versus log10 for genomic distances separating the contacting regions for the whole data set (black), intra-TAD contacts (red) and inter-TAD contacts (blue). (TIF) [file pgen.1006966.s006.tif]
